# Live-cell imaging of conidial anastomosis tube fusion during colony initiation in *Fusarium oxysporum*

**DOI:** 10.1371/journal.pone.0195634

**Published:** 2018-05-07

**Authors:** Smija M. Kurian, Antonio Di Pietro, Nick D. Read

**Affiliations:** 1 Manchester Fungal Infection Group, Institute of Inflammation and Repair, University of Manchester, Manchester, United Kingdom; 2 Departamento de Genetica, Universidad de Cordoba, Campus Rabanales C5, Cordoba, Spain; Oregon State University, UNITED STATES

## Abstract

*Fusarium oxysporum* exhibits conidial anastomosis tube (CAT) fusion during colony initiation to form networks of conidial germlings. Here we determined the optimal culture conditions for this fungus to undergo CAT fusion between microconidia in liquid medium. Extensive high resolution, confocal live-cell imaging was performed to characterise the different stages of CAT fusion, using genetically encoded fluorescent labelling and vital fluorescent organelle stains. CAT homing and fusion were found to be dependent on adhesion to the surface, in contrast to germ tube development which occurs in the absence of adhesion. Staining with fluorescently labelled concanavalin A indicated that the cell wall composition of CATs differs from that of microconidia and germ tubes. The movement of nuclei, mitochondria, vacuoles and lipid droplets through fused germlings was observed by live-cell imaging.

## Introduction

Conidial anastomosis tubes (CATs) are distinct types of cell protrusions that are different from germ tubes (GTs). CAT fusion results in the formation of interconnections between developing germlings during the early stages of colony development in filamentous fungi [1]. Within the developing colony, CAT fusion is believed to promote the transport of water and nutrients, which are commonly distributed heterogeneously within the natural environment [2]. Evidence has also been obtained that CAT fusion can facilitate horizontal gene transfer between strains to promote genetic variation [3].

*Fusarium oxysporum* is an asexual homothallic fungus with no reported sexual stage in its life cycle [4]. The *F*. *oxysporum* species complex includes many *formae speciales* which collectively infect over 120 different plant species [5, 6]. The genome possesses supernumerary chromosomes, which are thought to have been acquired by horizontal transfer during evolution [7]. Such studies have also revealed the versatile nature of the *F*. *oxysporum* genome, which is prone to gene transfer events leading to genetic variation within the species [8]. Horizontal gene transfer is an alternative to the common method of vertical chromosome transfer and meiotic recombination in eukaryotes [9], allowing the formation of recombinant strains through non-meiotic recombination [10]. Although evidence for horizontal gene transfer in filamentous fungi is increasing as a result of comparative genetic studies and genome sequencing, the mechanism by which heterokaryon formation occurs in nature is little understood [4, 7, 9, 11]. Vegetative hyphal fusion between mature colonies of incompatible species commonly results in death of the heterokaryotic cells formed [3, 12, 13]. Heterokaryon incompatibility has been shown to be suppressed following CAT fusion during colony initiation in the plant pathogen *Colletotrichum lindemuthianum* [3], but whether this occurs in other fungi is currently unknown. Vegetative incompatibility groups in *F*. *oxysporum* are based on heterokaryon formation by nitrate non-utilizing mutants, and around 80 putative HET domain proteins are present in the *F*. *oxysporum* genome [14]. In *F*. *oxysporum*, there is evidence for parasexual recombination between vegetatively incompatible strains, which is assumed to be mediated by hyphal anastomosis [15, 16].

Hyphal anastomosis in *Fusarium* was first described by Mesterhazy [17] who showed fusion between micro- and macroconidia of *F*. *oxysporum* f.sp. *medicaginis* and *F*. *graminearum*. Nuclear dynamics during fusion between microconidial germlings has been described in detail [18]. Vegetative hyphal fusion in general appears to be not required for plant infection as the *F*. *oxysporum* SO mutant, which is defective in hyphal fusion and can still infect tomato plants [19]. Shahi *et al*., [20] reported the formation of heterokaryotic cells by CAT fusion of two incompatible strains (Fol4287 and Fo47) and observed a dominance of Fol4287, with the nucleus of Fo47 being absent in the GTs emerging from heterokaryotic cells. However, heterokaryon incompatibility was not supressed since no evidence for nuclear fusion was obtained. Shahi *et al*., [14] produced deletion mutants in Fol4287 of a homolog of Vib-1, which is required for the expression of genes involved in heterokaryon incompatibility in *N*. *crassa* [21]. While these mutants exhibited an increased frequency of CAT fusion and heterokaryotic cells (cell compartments in which nuclei of different genotypes coexisted without fusing), heterokaryon incompatibility was not overcome in this mutant.

*Neurospora crassa* has been used as the main experimental model to study vegetative cell fusion in filamentous fungi [1, 22]. CAT fusion and its underlying signalling mechanisms have been extensively studied in this model organism in which over 50 mutants defective at different stages of CAT fusion have been characterised [1, 23, 24]. CAT fusion has been shown to be physiologically distinct and under separate genetic control from germ tube formation [25]. The basic stages of CAT fusion that have been described in *N*. *crassa* are: 1) CAT induction resulting in the emergence of CATs as small cell protrusions from either conidia or germ tubes; 2) CAT homing in which CATs exhibit chemotropic growth towards each other; and 3) CAT fusion in which the CAT tips make contact and the intervening cell walls become degraded and plasma membrane merger occurs between the fusing cells [2, 26]. CAT fusion establishes cytoplasmic connections between germlings enabling the movement of nuclei and organelles through them [27, 28].

The conditions, which induce CAT fusion *in vitro* have been shown to vary in different fungi. In *N*. *crassa*, for example, it occurs in Vogel’s minimal medium within 4–6 h of inoculation [26] whereas in the plant pathogen *C*. *lindemuthianum*, CAT fusion is inhibited by growth medium and only occurs in water after incubation for 72 h [3, 28].

The aims of this study on CAT fusion in *F*. *oxysporum* were to: (1) identify the optimal conditions that favour CAT fusion in liquid media; (2) develop a reproducible method for routine quantitative live-cell imaging of CAT fusion; (3) provide a detailed cytological analysis of CAT fusion using live-cell imaging; and (4) determine whether CAT fusion facilitates the movement of nuclei and other organelles between germlings.

## Materials and methods

### Strains, media and culture conditions

The *Fusarium oxysporum* f. sp. *lycoperisici* wild-type strain 4287 (FGSC 9935) and its derivatives expressing either sGFP [29], histone H1-GFP or H1-mCherry [18] were used in this study. Stock cultures of the different *F*. *oxysporum* strains were prepared as microconidial suspensions at a concentration of 1 x 10^7^ spores per ml, and stored in a final volume of 30% glycerol at -80°C. Potato dextrose broth (PDB) was prepared from commercially available baking potatoes, so that a final volume of 5 L contained diluted potato broth from 1 kg potatoes with 2% D-glucose (Fisher Scientific). Liquid minimal medium was prepared by dissolving the following ingredients in 1 L of tap water: 1 g KH_2_PO_4_; 0.5 g MgSO_4._7H_2_O; 0.5 g KCl; 2 g NaNO_3_; 20 g glucose and 200 g sucrose. Cultures for harvesting microconidia were grown in 100% PDB at 28°C with shaking at 180 rpm. Microconidia were harvested from 5 to 10 days old liquid cultures by filtering the cultures through 2 layers of sterile Miracloth (Calbiochem). This was followed by two washing steps of the cultures with sterile distilled water in centrifugation tubes at 16770 x g for 10 min at 25°C. Spore suspensions of defined concentrations were produced from the pelleted spores after cell counting using a haemocytometer (Fuchs Rosenthal chamber slide).

All culture optimisation experiments and cytological studies were conducted in the wild type strain (4287) of *F*. *oxysporum* unless specified.

### Adhesion, germination and CAT assays

CAT fusion assays were set up in 8-well borosilicate slide culture chambers (Nalge Nunc, Rochester, NY). 300 μl of a microconidial spore suspension was added to each well and incubated at 25°C for 12 h in continuous light. A 1% dilution of the 100% PDB with 25 mM NaNO_3_ was used as the liquid medium for CAT fusion and spore germination assays unless otherwise specified. Any spore in which the oval symmetry had been broken due to the emergence of a tube-like protrusion was counted as germinated. The percentage of spores undergoing CAT fusion was determined by counting the spores or spore germlings that participated in CAT fusion. For example, a CAT fusion formed between two spores or between a spore and a germling or between two germlings was scored as 2 in each case. Spores that formed germ tubes alone and were not involved in cell fusion were counted separately and were then added to the number of CAT fusions to give the total number of germinated spores. This is because spore germination can be considered as involving the formation of a germ tube and/or a CAT. CAT fusion and spore germination were quantified from a minimum of 350 cells counted from microscopic images. All experiments were performed at least three times unless otherwise stated.

#### Microconidial adhesion assays

Adhesion was scored from short movies of 1 min with an image captured every second. The germlings that stopped exhibiting Brownian movement and remained attached to the base of the chamber slide were scored as adhered whilst the ones which continued to move were scored as non-adhered.

### pH measurements

The pH of the standard growth medium (1% PDB supplemented with 25 mM NaNO_3_) used for the spore germination and CAT fusion assays was naturally at pH 5.5 and did not need adjusting. However, for experiments in which the influence of the external pH on spore germination and CAT fusion was analysed, the growth medium was adjusted with an Orion 710A+ pH meter (Thermoscientific) using 10M KOH, 5M NaOH and 16% HCl. The buffers used for adjusting the pH of media to the desired pH value for experiments with buffered pH media are shown in Table 1. 10 ml of the appropriate filter-sterilized buffer was mixed with 90 ml of autoclaved media (1% PDB or 1% PDB supplemented with 25 mM NaNO_3_) to produce a final 100 mM concentration of the buffer in the medium. Prior to using the buffered media for experiments, their pH was double-checked in small volumes separated from the buffered media to be used.

**Table 1:** Buffers used for adjusting the pH of growth media.

Buffer used	pH
1 M Glycine-HCl (pH adjusted to 3.5 with concentrated HCl)	3.5
1 M Glycolic acid (pH adjusted to 5 with 10 M NaOH)	5.0
1 M MES (pH adjusted to 6.5 with 10 M NaOH)	6.5
1 M Tris-HCl (pH adjusted to 7.5 with concentrated HCl)	7.2
1 M Tris-HCl (pH adjusted to 8.3 with concentrated HCl)	8.0

A pH glass microelectrode (Orion 9810BN, Fischer Scientific UK Ltd) was used in experiments to determine the influence of different medium supplements added to 1% PDB in the 8-well, slide culture chambers in which spore germination and CAT fusion were analysed. The initial pH and final pH after 12 h of incubation were measured in each experiment. Data was collected from three different experiments. Datasets were also generated from experiments in the absence of fungal cells, which were used as controls. These datasets were used to normalise the values obtained with the spore germlings present to generate the mean values of the change in media pH over the 12 h of incubation. The buffers and buffered media were stored at 4°C.

### Microscopy

#### Live-cell imaging of spore germination and CAT fusion *in vitro*

Live-cell imaging of spore germination and CAT fusion was performed in 8-well cell culture chambers. Brightfield light microscopy and differential interference contrast (DIC) microscopy were performed using a Nikon Eclipse TE 2000E inverted microscope with a 60x/1.20 NA water immersion plan apo objective. A Hammamatsu Orca-ER CCD camera and Metamorph software (Universal imaging) was used for image acquisition. Further image analysis was done using Image J software (rsbweb.nih.gov/ij). Confocal microscopy was performed using a Leica TCS SP8 inverted microscope with a white light laser. The white light laser used was set at an excitation wavelength according to the optimal emission peak obtained after a λ-scan for the different fluorophores used. LASAF software was used for image processing. The fluorescent dyes used for labelling organelles are listed in Table 2 and the excitation and emission peaks used for each fluorescent probe are shown in Table 3. In case of Calcofluor white, a 20 min incubation period at room temperature was required before imaging.

**Table 2:** Fluorescent dyes used for confocal live-cell imaging.

Stain	Organelle labelled	Working concentrations	Source
Alexafluor conjugate of Concanavalin A (Con A)	cell wall	10 μg/ml	Sigma
Alexafluor conjugate of Wheat germ agglutinin (WGA)	cell wall	10 μg/ml	Molecular Probes
Calcofluor white (CFW)	cell wall	83 ng/ml	Sigma
cDFFDA	vacuoles	6.45 μg/ml	Sigma
Mitotracker Red	mitochondria	0.36 ng/ml	Molecular Probes
Nile red	lipid bodies	1 μg/ml	Sigma
DAPI	nuclei	0.083 ng/ml	Sigma

**Table 3:** Excitation and emission peaks of labels/fluorophores used.

Label/fluorophore	Excitation wavelength (nm)	Emission wavelength (nm)
sGFP	488	508–551
mCherry	569	609–681
Con A	488	512–596
WGA	488	510–569
CFW	405	424–476
cDFFDA	488	505–558
Mitotracker Red	581	622–689
Nile red	520	571–637
DAPI	405	432–462

## Results

### Media composition influences microconidial germination, CAT fusion and germling adhesion

**Fig 1:**
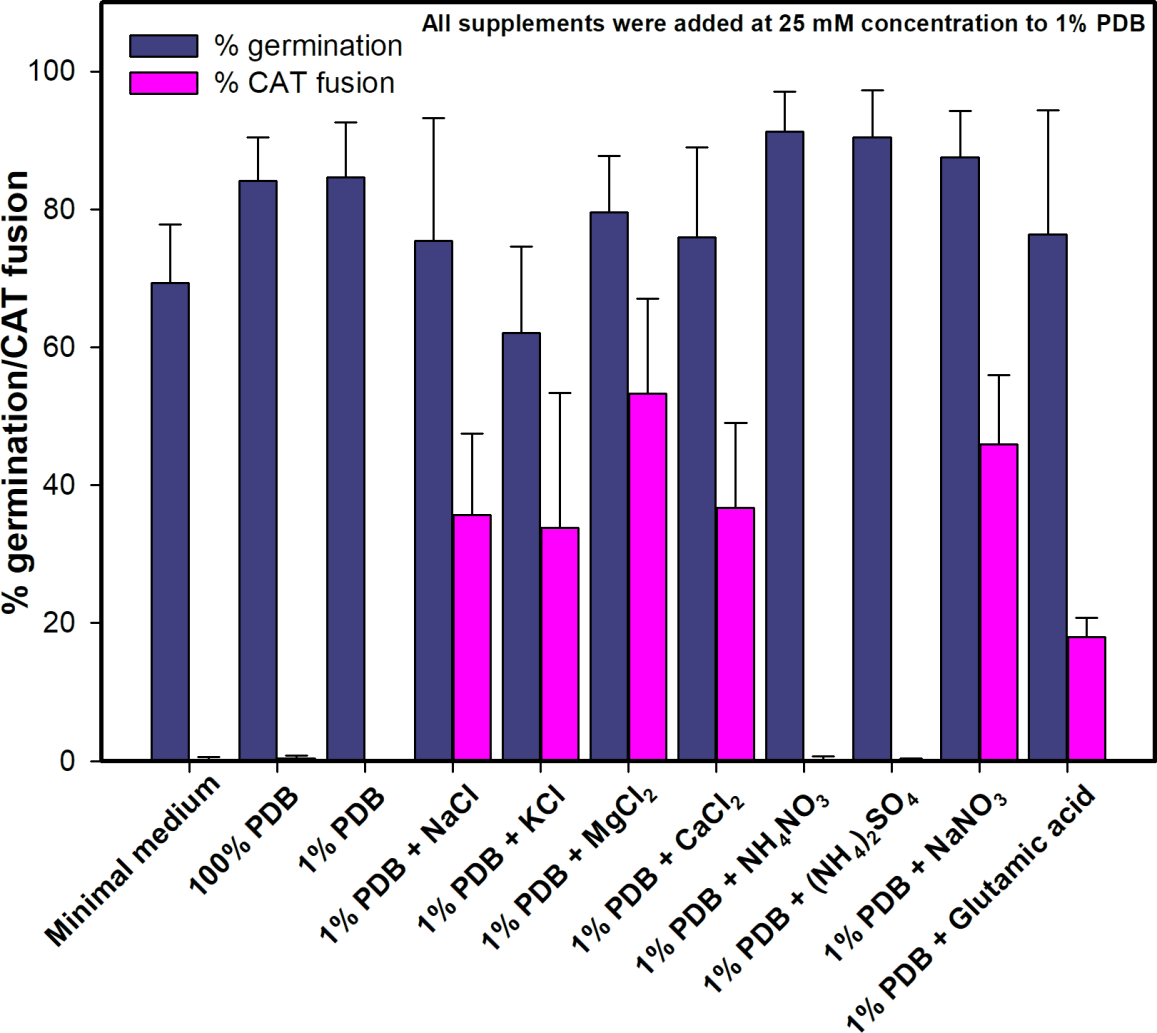
Germination and CAT fusion of microconidial and macroconidial germlings in different media. CAT fusion was only observed in 1% PDB supplemented with various chemicals/nutrients at 25 mM (NaNO_3_, CaCl_2_, NaCl, MgCl_2_, KCl or monosodium salt of glutamic acid). 1x10^6^ spores/ml used.

**Fig 2:**
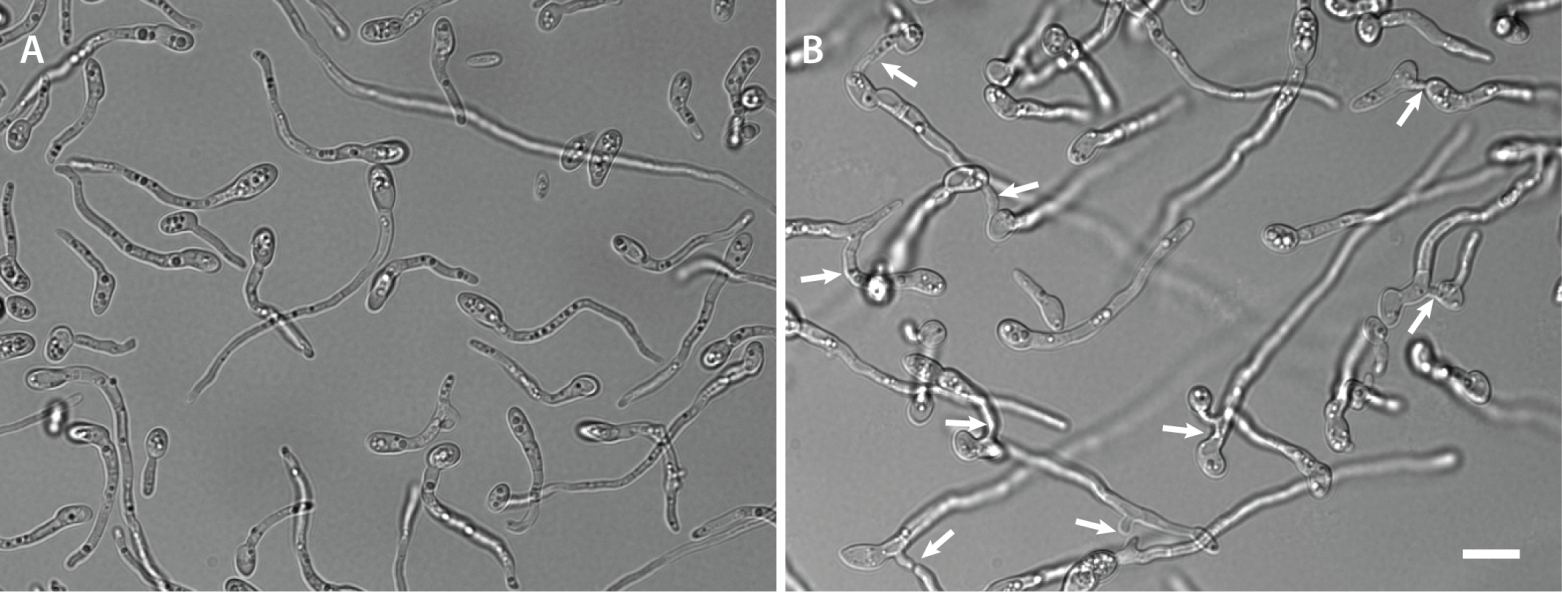
**(A) CAT fusion does not occur in microconidia germinated in 1% PDB alone** (also see S1 Movie). **(B) CAT fusion (arrows) occurs in 1% PDB supplemented with 25 mM NaNO**_**3**_ (also see S2 and S3 Movies). Both samples were imaged 12 h post inoculation. 1x10^6^ spores/ml used. Scale bar = 10 μm.

**Fig 3:**
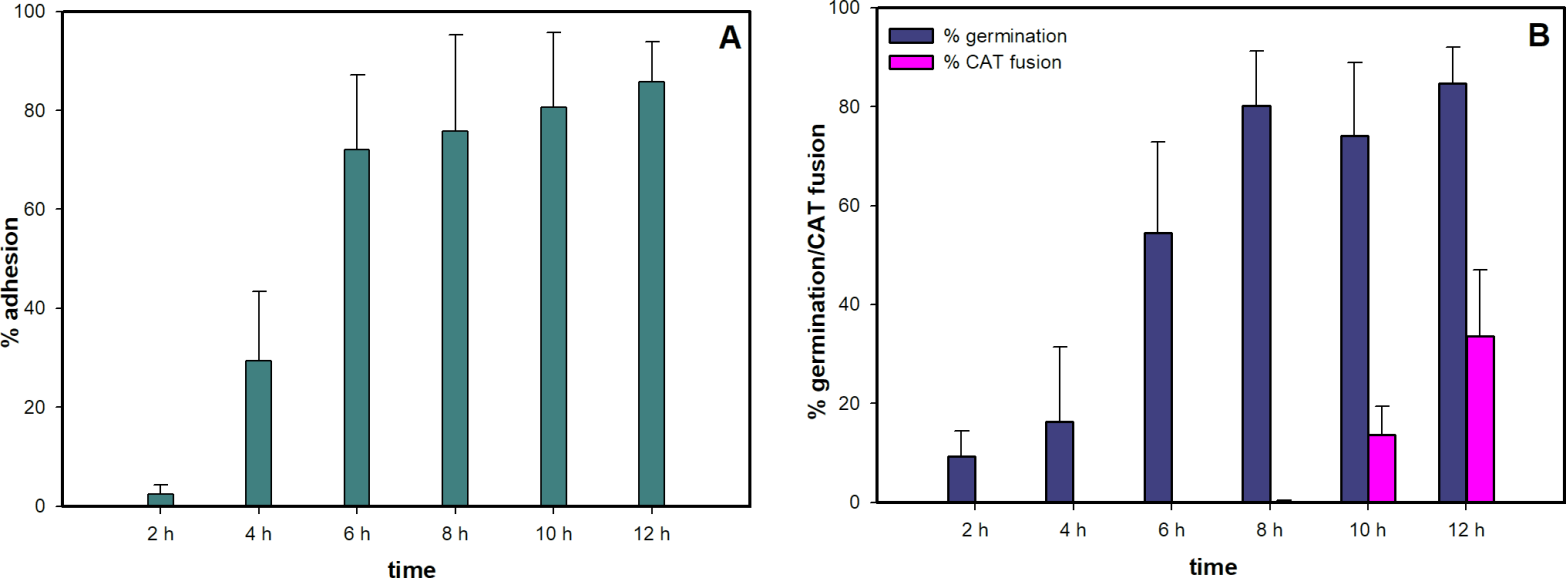
(**A) Rate of adhesion of microconidia and microconidial germlings in 1% PDB + 25 mM NaNO**_**3**_. **(B) Rate of microconidial germination and CAT fusion in 1% PDB + 25 mM NaNO**_**3**_. 1x10^6^ spores/ml were used.

Inoculating a rich medium (100% PDB) or a minimal liquid medium with microconidia failed to induce CAT fusion in *F*. *oxysporum* (Fig 1). However, CAT fusion occurred in 1% PDB supplemented with one of a number of different chemicals (NaNO_3_, CaCl_2_, NaCl, MgCl_2_, KCl or glutamic acid monosodium salt). In 1% PDB medium alone, the microconidia underwent adhesion-independent germination. The microconidia and germlings exhibited Brownian movement in the liquid medium and did not adhere to the borosilicate glass substratum of the slide culture chambers (Fig 2A; S1 Movie). However, when 1% PDB was supplemented with one of a wide range of chemicals at 25 mM concentration (NaNO_3_, CaCl_2_, NaCl, MgCl_2_, KCl, NH_4_NO_3_, (NH_4_)_2_SO_4_ or glutamic acid monosodium salt), adhesion of the germlings to the glass substrate occurred. The percentage of adhered microconidia and microconidial germlings increased from 2 to 12 h following inoculation (Figs 2B and 3A; S2 and S3 Movies). In 1% PDB supplemented with NaNO_3_ a few microconidia started to form germ tubes before adhesion took place, but most germ tubes were formed after adhesion had occurred. Germ tube formation was initiated within the first 2 h post inoculation, and CAT fusion started after ~ 8 h when 80% of the microconidia had germinated (Fig 3B). Under these conditions, microconidia only produced single germ tubes (Fig 2A and 2B). Interestingly, CAT fusion did not occur in 1% PDB in the presence of NH_4_NO_3_ or (NH_4_)_2_SO_4_, even though adhesion and 80–90% germination had occurred in media containing these supplements (Fig 1). The adhesion of germlings incubated in 1% PDB supplemented with NaNO_3_ was found to be very persistent after incubation for 12 h, because they could not be removed by adding water droplets using a pipette (S4 Movie).

The frequency of CAT fusion in 1% PDB supplemented with different nutrients/chemicals differed depending on the nutrient/chemical used (Fig 1). Optimisation of the composition of the liquid medium producing maximum levels of CAT fusion was performed using different concentrations of NaNO_3_. 1% PDB medium supplemented with 25 mM NaNO_3_ was selected and routinely used in subsequent studies of CAT fusion *in vitro*, unless otherwise stated. Approximately 40% of CAT fusion was reproducibly obtained in this medium after 12 h of inoculation (Fig 1). From the results presented in this section, it was concluded that germ tube formation precedes CAT formation, and that microconidial adhesion is a prerequisite for CAT formation and CAT homing leading to CAT fusion.

To examine cell adhesion in more detail, microconidia were incubated in 1% PDB for 7.5 h in a slide culture chamber. At this time point most microconidia had germinated to form germ tubes, but none of them had adhered to the underlying glass substratum. Two 20 μl drops of 50 μg/ml NaNO_3_ were then added to these germlings in liquid growth medium to give a final concentration of ~ 25 mM NaNO_3_ in the slide culture chamber well. Live-cell imaging showed that addition of NaNO_3_ resulted in the germlings becoming immediately immobilised as they adhered to the glass (S5 Movie). Furthermore, when these germlings were incubated for an additional 4.5 h (to make the total time of incubation 12 h), significant CAT fusion had occurred between the adhered germlings. This provides further evidence that CAT fusion is dependent on cell adhesion. Furthermore, microconidia underwent CAT fusion more rapidly (< 4.5 h) when NaNO_3_ was added at 7.5 h post-inoculation (S5 Movie) than when NaNO_3_ was provided at the time of inoculation, in which case CAT fusion was first recorded at 8 h (Fig 3B). This suggests that microconidia require a certain period following inoculation to reach developmental competence to respond to the adhesion stimulus to form CATs.

In summary, microconidia of *F*. *oxysporum* exhibited two different patterns of germination depending on the nutrient status of the medium: (1) adhesion-independent germination in which only GTs are formed and no fusion is detected (Fig 2A; S1 Movie); and (2) adhesion-dependent germination in which GTs and CATS are formed and the CATs undergo fusion (Fig 2B; S3 Movie).

### Influence of temperature on CAT fusion

**Fig 4:**
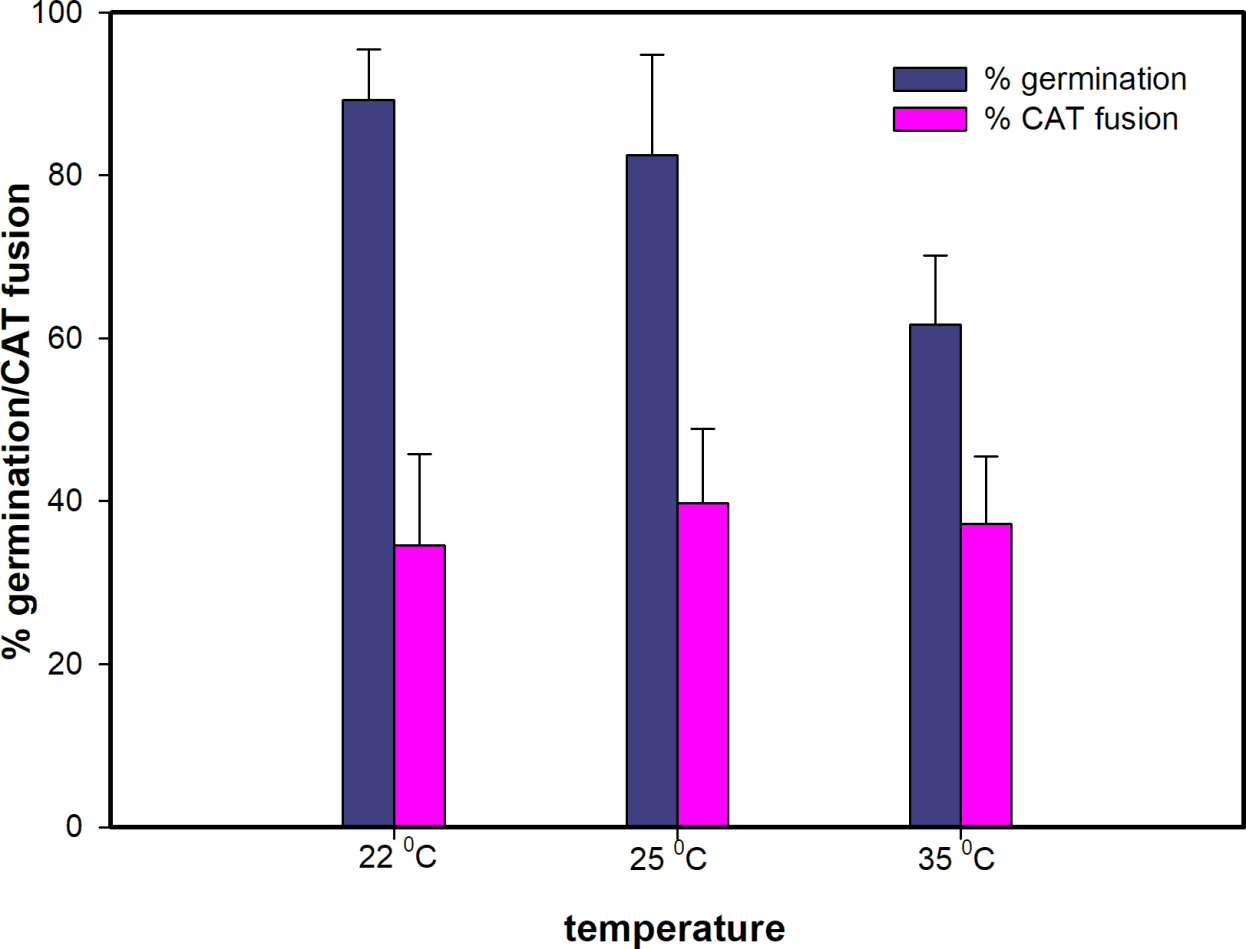
Influence of temperature on microconidial germination and CAT fusion in 1% PDB + 25 mM NaNO_3_. With the exception of a ~15% reduction in germination at 35°C, the three temperatures tested had no significant influence on the percentage of spore germination or CAT fusion. 1x10^6^ spores/ml were used.

The influence of different incubation temperatures (22°C, 25°C and 35°C) on microconidial germination and CAT fusion was assessed. Germination was significantly inhibited at 35°C (*p* < 0.05 from a t-test in which the germination at 25°C and 35°C were compared from 4 experiments; *p* < 0.001 when germination at 22°C and 35°C were compared from 3 experiments) whilst CAT fusion occurred to a similar extent at all three temperatures tested (Fig 4). As a result, quantification of microconidial germination and CAT fusion *in vitro* were routinely performed at 25°C.

### Influence of cell density on CAT fusion

**Fig 5:**
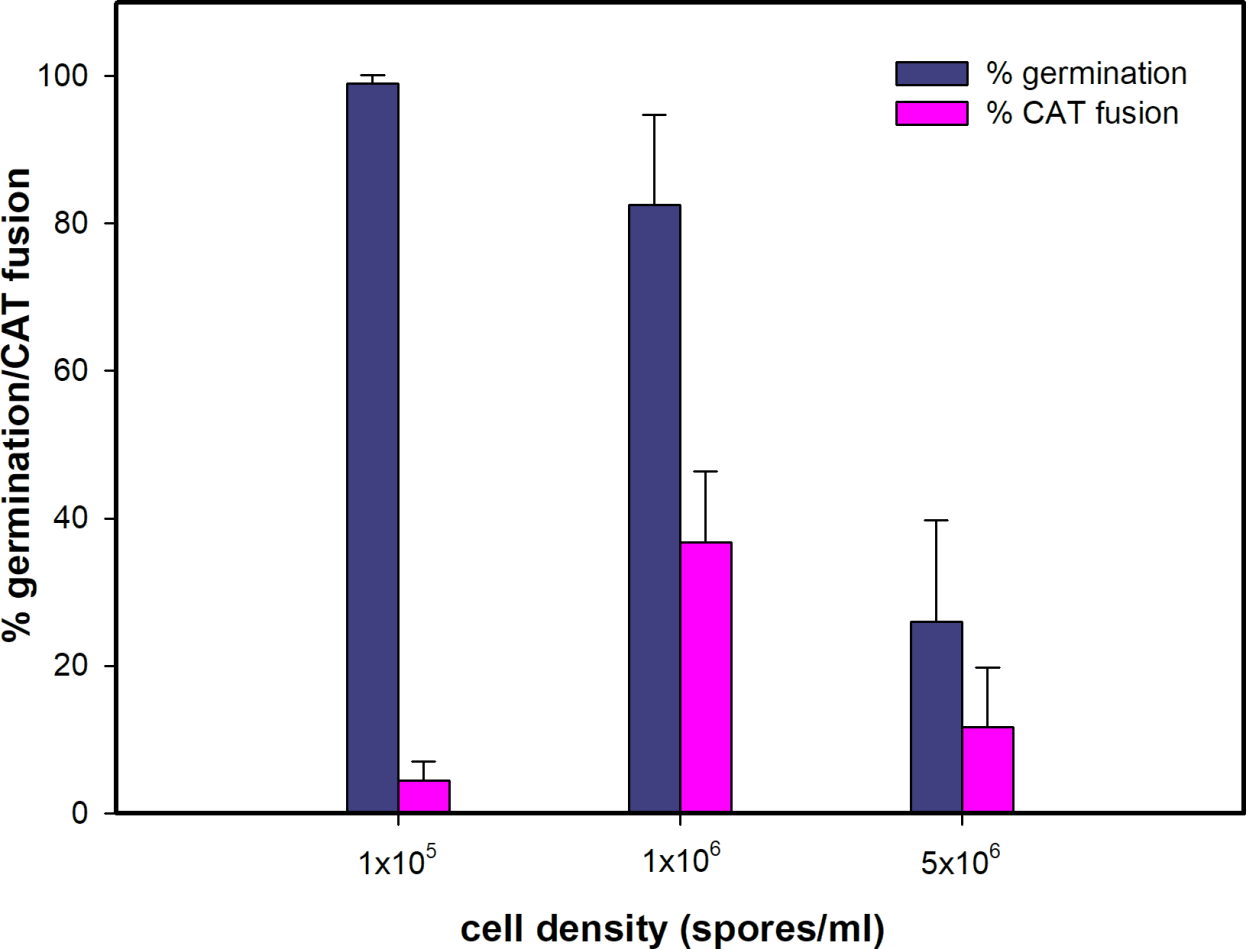
Effect of microconidial density on germination and CAT fusion in 1% PDB + 25 mM NaNO_3_. 1 x 10^6^ spores/ml provided optimal CAT fusion.

Three different microconidial densities, 1 x 10^5^, 1 x 10^6^ and 5 x 10^6^ per ml, were assessed in 8-well slide culture chambers for spore germination and CAT fusion. A density of 1 x 10^5^ per ml was found to be optimal for germination, while 1 x 10^6^ per ml was optimal for CAT fusion (Fig 5). Since the focus of the current study was on CAT fusion, all experiments were routinely performed at 25°C with 1 x 10^6^ spores/ml.

### Influence of pH on CAT fusion

**Fig 6:**
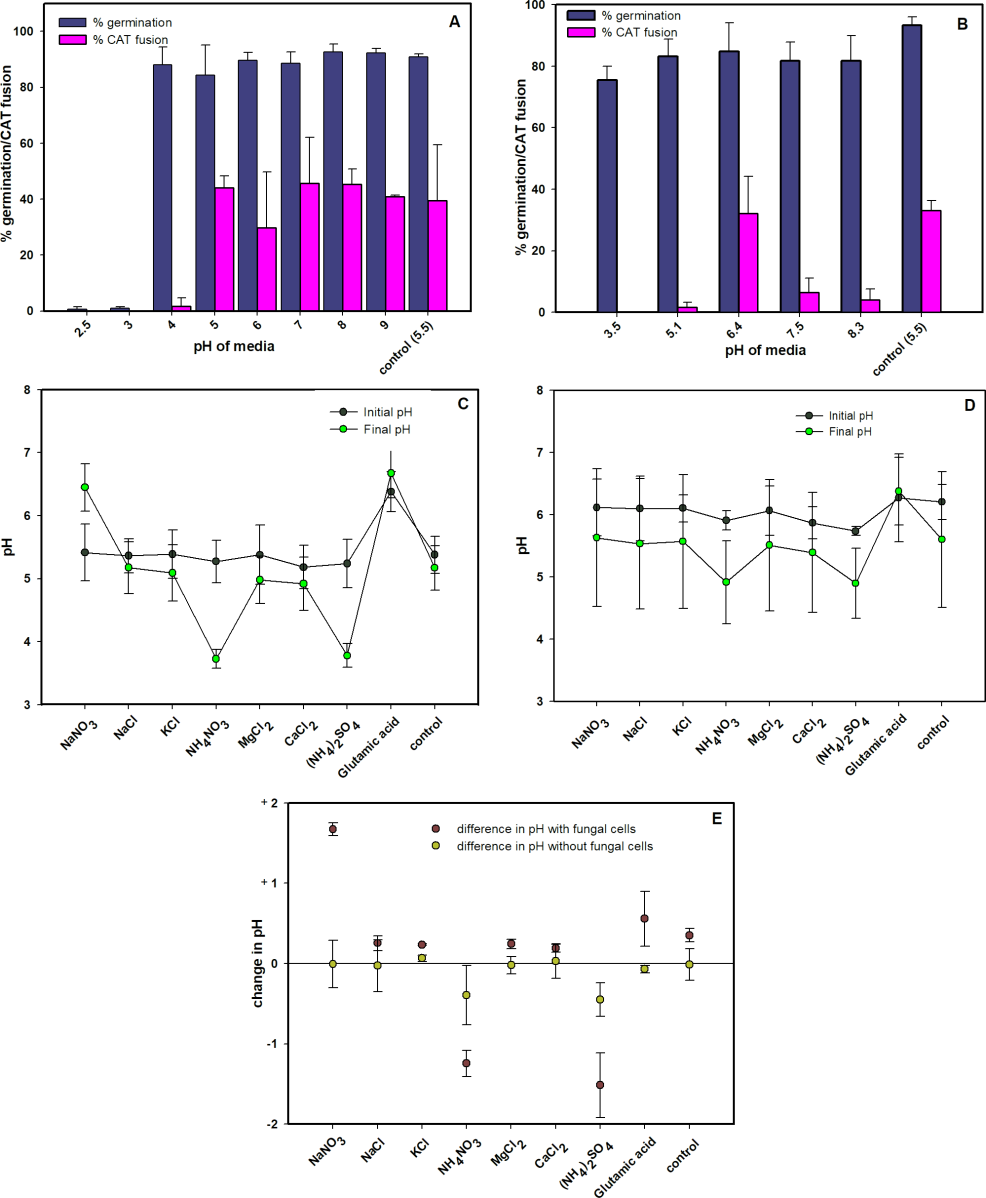
Influence of pH on germination and CAT fusion. (A) Influence of pH on microconidial germination and CAT fusion at 12 h post inoculation in unbuffered media (1% PDB + 25 mM NaNO_3_) whose pH was adjusted to different pH values. The control represents the medium in which the pH was not adjusted. (B) Influence of pH on microconidial germination and CAT fusion at 12 h post inoculation in buffered media (1% PDB + 25 mM NaNO_3_). The control represents the medium in which the pH was not adjusted. (C) Initial and final pH of unbuffered 1% PDB with different supplements that promote CAT fusion, inoculated with 1x10^6^ spores/ml and incubated for 12 h. The control was medium lacking supplements. (D) Initial and final pH of unbuffered 1% PDB with different supplements, incubated in the absence of fungal cells for 12 h. The control was medium lacking supplements. (E) Change in pH values of media with different nutrients after 12 h incubation in the presence and absence of fungal cells. The results shown in this Figure are plotted from data shown in Fig 6C and 6D. The control is medium lacking supplement. 1x10^6^ spores/ml were used.

CAT fusion in unbuffered, pH-adjusted medium (1% PDB + 25 mM NaNO_3_) was found to occur at a similar level between pH 5 and 9, but was greatly or completely inhibited at pH values of 4 or below (Fig 6A). Microconidial germination occurred at a similar level over a slightly wider pH range (pH 4–7) and was greatly or completely inhibited at pH values < 4 (Fig 6A). The effect of buffering the medium to pH values 3.5, 5.1, 6.4, 7.5 and 8.3 was also tested. The results of these experiments were similar to those obtained with unbuffered media (Fig 6B). As a result, experiments involving the quantification of germination and CAT fusion were routinely performed in unbuffered medium (1% PDB + 25 mM NaNO_3_) with an initial pH of 5.5 (Fig 6A).

We next asked whether the pH of the medium changed over 12 h of incubation in the presence of the fungus. In addition, a comparison was made of the initial and final pH values between the standard medium (1% PDB + 25 mM NaNO_3_) and unbuffered 1% PDB with different chemical supplements tested previously (see Fig 1). This experiment showed significant differences in the pH values of some of the media at the beginning and end of the 12 h incubation period (Fig 6C). As a control, the experiment was performed in the absence of fungal cells (Fig 6D). These results showed that incubation in the presence of the fungal cells for 12 h increased the pH values of all media except for those supplemented with NH_4_NO_3_ or (NH_4_)_2_SO_4_ in which the pH dropped below pH 4 after 12 h incubation (Fig 6). The standard medium (1% PDB + 25 mM NaNO_3_) routinely used in our assays induced the highest pH increase among all the media tested (Fig 6E). The final pH of the media correlated with the presence or absence of CAT fusion previously observed in these media (Fig 1) and in the standard medium (1% PDB + 25 mM NaNO_3_) in which the pH had been adjusted to different values (Fig 6B).

### Different types of CAT fusion

**Fig 7:**
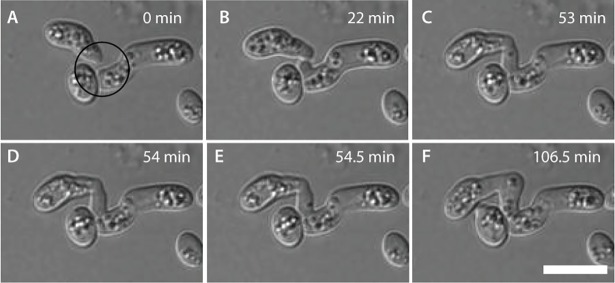
Three stages of CAT fusion in *F*. *oxysporum*: CAT formation, CAT homing and CAT fusion. (A) CAT formation indicated by the formation of cell projections (circle) from the tip and side of two GTs (at 0 min). (B) CAT homing where the two cell projections (CATs) grow towards each other (at 22 min). (C-F) CAT fusion where the CATs attach and the cell walls in between break down establishing a cytoplasmic connection between the two germlings (53 min to 106.5 min). 1x10^6^ spores/ml used. Scale bar = 10 μm.

**Fig 8:**
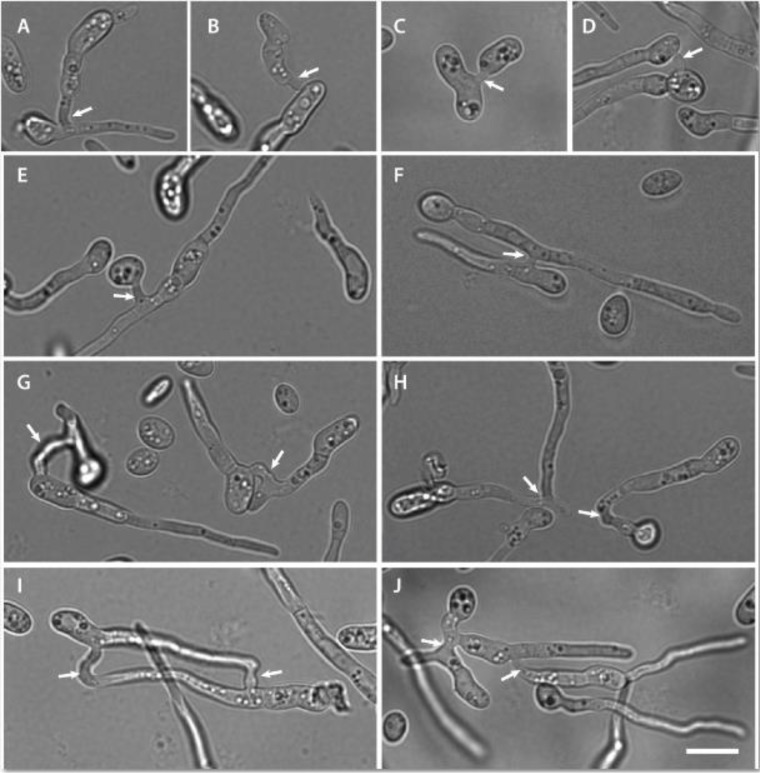
Different types of CAT fusion observed between germlings. (A, F, H and I) CAT fusion between two GTs. (C) CAT fusion between two conidia that have not produced GTs. (D) CAT fusion between two conidia that have already produced long GTs. (B, E, G) CAT fusion between a GT and a conidium. CAT fusion resulted from tip-to-tip fusion of CATs formed from growing germ tubes (H), tip-to-side fusion of a CAT formed from a growing germ tube to a spore (A, B and I), and side-to-side fusion of CATs formed from adjacent germ tubes (F, G). Arrows point to region of CAT fusion. 1x10^6^ spores/ml were used. Scale bar = 10 μm.

Different types of CAT fusion were observed in the CAT fusion medium (1% PDB + 25 mM NaNO_3_) all of which involved positive tropisms of CATs towards each other (Figs 7 and 8): between two germ tubes; between two ungerminated spores; or between a germ tube and a ungerminated spore. Multiple fusion events between two germlings and fusion between multiple germlings were also observed (Fig 8I and 8J). In some cases, CAT fusion was observed between cells that were immediately adjacent to each other (e.g. S1 Movie). As reported previously [18], CAT fusion resulted either from tip-to-tip fusion of CATs formed from growing GTs, tip-to-tip fusion between spores, tip-to-side fusion of CATs formed from a growing GT to a spore, and side-to-side fusion of CATs formed from adjacent GTs (Fig 8). Tip-to-tip fusion resulted in the fused hyphae subsequently growing from the fused region as a single GT (Fig 8H).

In *N*. *crassa*, a key morphological feature differentiating CATs from GTs is the difference in width [25]. In *F*. *oxysporum*, CATs were generally thinner than GTs, the average width of CATs being 1.73 ± 0.52 μm as compared to 2.45 ± 0.45 μm for GTs (n = 100) (*p* = 0 from a t-test in which the widths of 100 CATs and 100 GTs were compared).

### Stages of CAT fusion

CAT fusion occurred after GT formation. Microconidia started to germinate 2–4 h post incubation at 25°C in 1% PDB supplemented with NaNO_3_, but CAT formation, recognizable as CAT fusion, started to occur at a later stage (~ 9 h of incubation) in suitable media (Fig 4). Although 80% of microconidia had germinated (formed GTs alone) at 8 h, hardly any CAT fusion was observed at this time.

**Fig 9:**
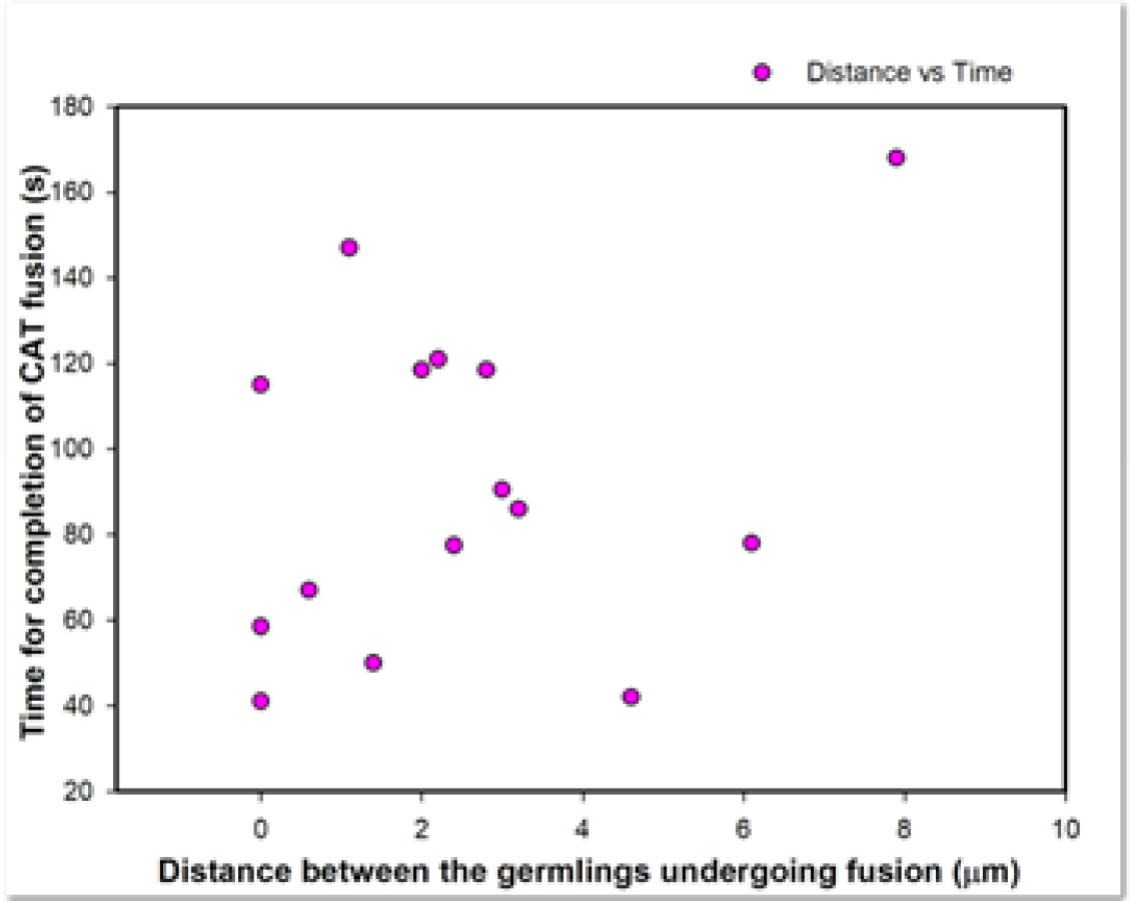
Scatterplot of the time required for CAT fusion from the initial appearance of the CAT projection to completion of fusion. Data obtained from 15 movies. 1x10^6^ spores/ml were used.

The three main stages of CAT fusion described in *N*. *crassa* (CAT formation, homing and fusion) [25] were also observed in *F*. *oxysporum* grown in liquid medium (Fig 7;S6 Movie). In this time course study, at time 0 min a small projection emerges from the side of the tip of the lower germ tube, and a smaller projection from the tip of the upper germ tube. The two incipient CATs grow towards each other (0 to 53 min), the tips of the CATs attach (53 min) and finally the intervening cell walls of the two CATs are degraded resulting in cytoplasmic continuity between the two hyphae (54.0–54.5 min). In S6 Movie, refractile structures (later identified as lipid droplets–see below) can be observed to move through the fused CATs from one germling to the other. The duration for the entire fusion process from the initial appearance of CAT projections to the completion of fusion, as determined from the analysis of 15 movies, varied between 42 min and 168 min. A scatterplot of the data revealed that there was no correlation between the distance between germlings and the time required for completion of CAT fusion (Fig 9). Unequal growth was observed between the two CATs in 9 of the 15 time courses analyzed, resulting in one CAT grown longer than the other. In 6 samples the two CATs had approximately equal length. The initial distance between the spores/germlings that ultimately fused varied between 0 μm (S7 Movie), when the fusing cells were immediately adjacent to each other, and ~ 8 μm (Fig 9). In three instances, the germlings initially touched each other and then became displaced as one or both CATs grew between the cells, resulting in the germlings being pushed apart. Although it was not possible to visualise in this time-course, cytoplasmic continuity between the two germlings was likely achieved at some point during the germling displacement process (S7 Movie). An analysis of 46 instances of CAT formation, homing and fusion from 10 different time courses showed that 80% (n = 46) had two CATs growing towards each other while 20% (n = 46) had just one CAT formed which grew towards the other partner cell (spore or GT) and ultimately fused (n = 46).

### Differences in cell wall composition of CATs and germ tubes

**Fig 10:**
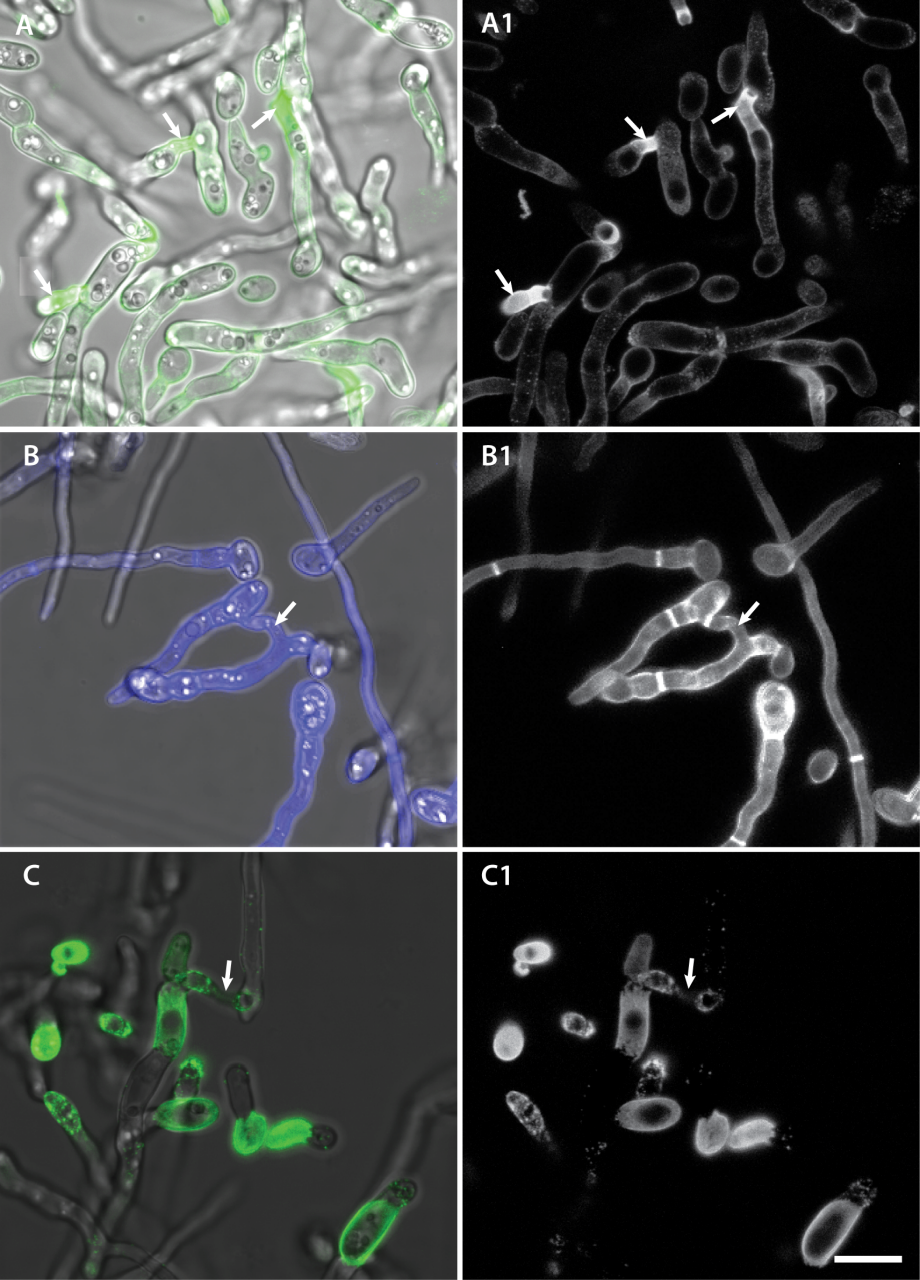
The cell wall composition of CATs is different from that of germ tubes and microconidia. (A, A1) Staining with Con A conjugated to Alexa fluor 488 showed that the regions of CAT fusion exhibit brighter staining than germ tubes. (B, B1) Staining with CFW showed no differential staining between CATs and germ tubes. (C, C1) Staining with WGA tagged with Alexa fluor 488 showed strong staining of microconidia, spot-like staining of germ tubes behind the growing tip but no staining of CATs. A, B and C are overlay images of the fluorescence and brightfield channels whilst A1, B1 and C1 shows single channel images of fluorescence images alone. Arrows point to region of CAT fusion. Scale bar = 10 μm.

Fluorescently tagged lectins were used to identify cell wall components on the surfaces of GTs and CATs. An Alexa fluor 488 conjugate of concanavalin A (Con A), that binds to α-mannopyranosyl and α-glucopyranosyl residues of α-mannans and α-glucans [30], exhibited significantly brighter staining of CAT cell walls, especially in CATs that were undergoing fusion or had recently fused. In contrast, the GT cell walls stained only weakly (Figs 10A, 10A1 and 11; S8 Movie). CFW, which is a non-specific fluorochrome that labels β-1,3- and β-1,4-linked glucans such as chitin throughout cell walls and septa [30, 31], uniformly stained the cell walls of conidia, GTs and CATs (Fig 10B and 10B1). An Alexa fluor 488 conjugate of WGA, which binds to N-acetyl glucosamine (i.e. chitin) and sialic acid residues on the surface of the cell wall [30], did not stain the cell walls of CATs whilst spotty stained regions were observed behind the growing tips of germ tubes (Fig 10C and 10C1).

**Fig 11:**
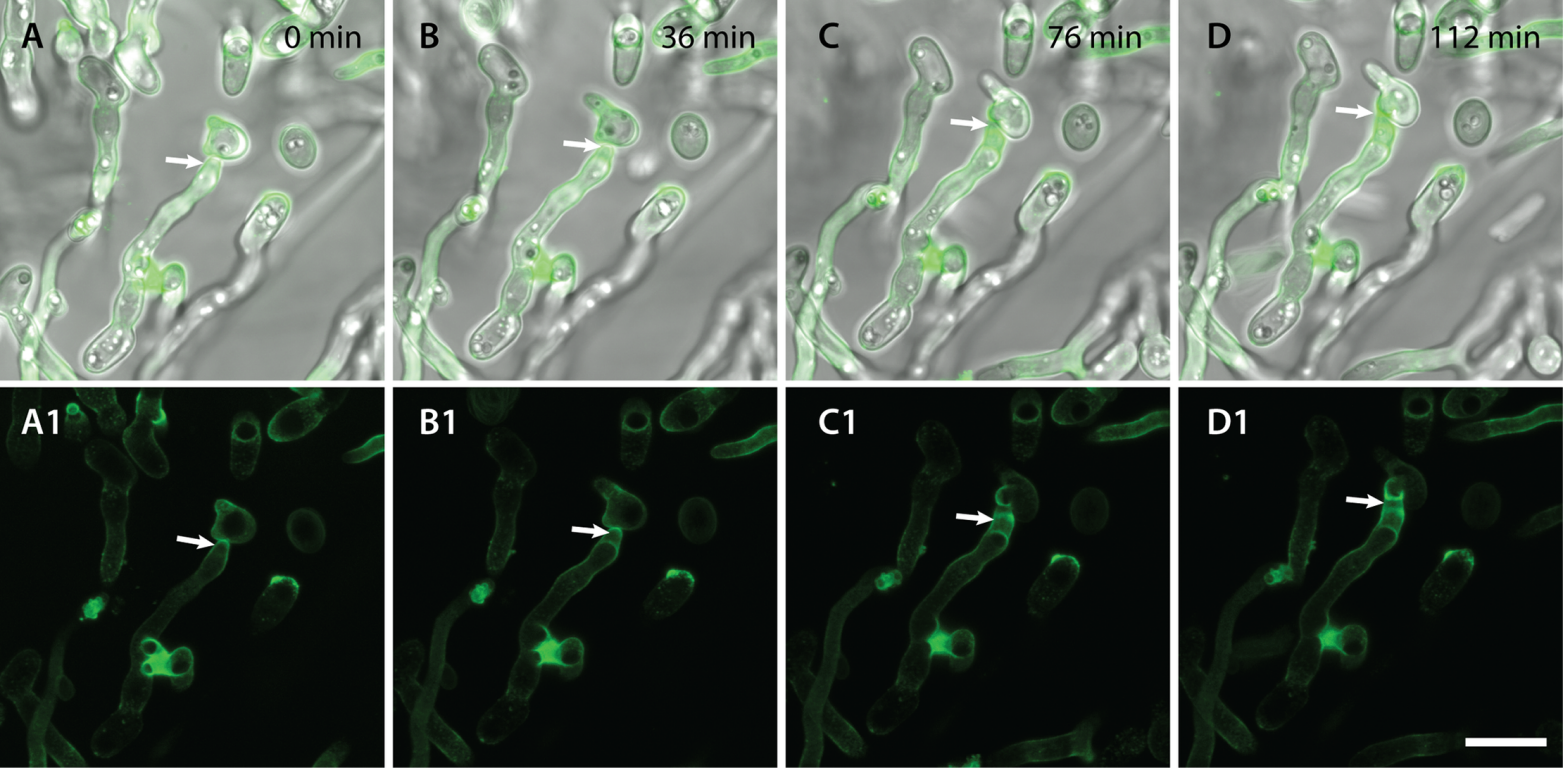
Time course showing bright staining of sites of CAT fusion with Con A conjugated to Alexa fluor 488. The CAT fusion event indicated by an arrow is occurring between a CAT emerging from the tip of a germ tube and a CAT from an adjacent microconidium (see Movie 4.1). A-D are overlay images of brightfield and fluorescence channels whilst A1-D1 are the corresponding images of fluorescence channel alone. Scale bar = 10 μm.

**Fig 12:**
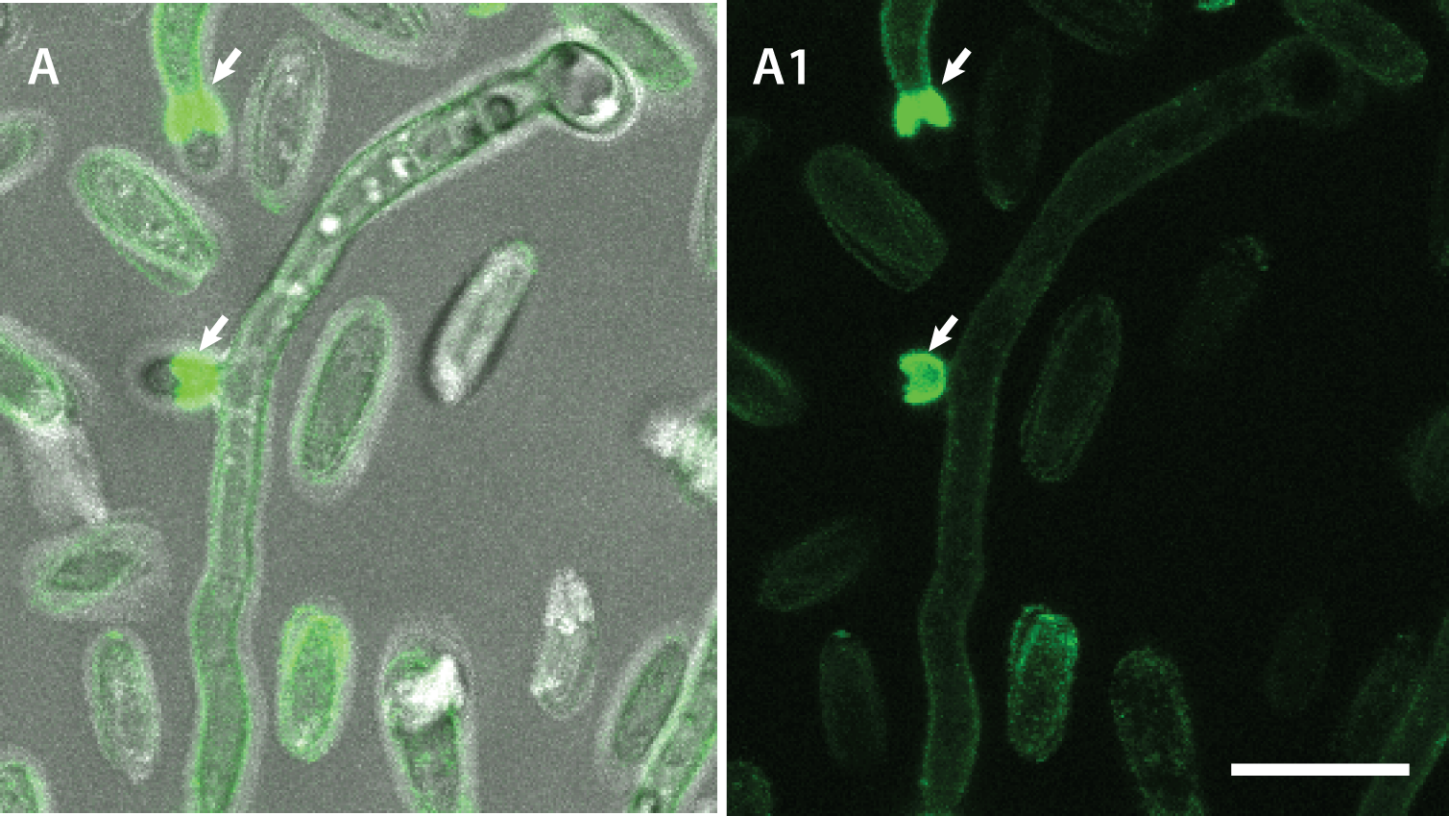
Con A staining of *F*. *oxysporum* germlings. (A, A1) Con A conjugated to Alexa fluor 488 brightly stains the basal regions of conidiogenous cells (indicated by arrows) from which microconidia are formed along the sides of hyphae. A is an overlay of the fluorescence and brightfield channels while A1 shows the fluorescence channel alone. Scale bar = 10 μm.

Con A conjugated to Alexa fluor 488 brightly stained the basal regions of conidiogenous cells from which microconidia are formed along the sides of hyphae (Fig 12). Cell walls of ungerminated spores were uniformly stained with the Con A-Alexa fluor 488 conjugate (Figs 10A, 10A1, 11 and 13), with the WGA-Alexa fluor 488 conjugate (Figs 10C, 10C1, 13B and 13B1) and with CFW (Fig 10B and 10B1).

**Fig 13:**
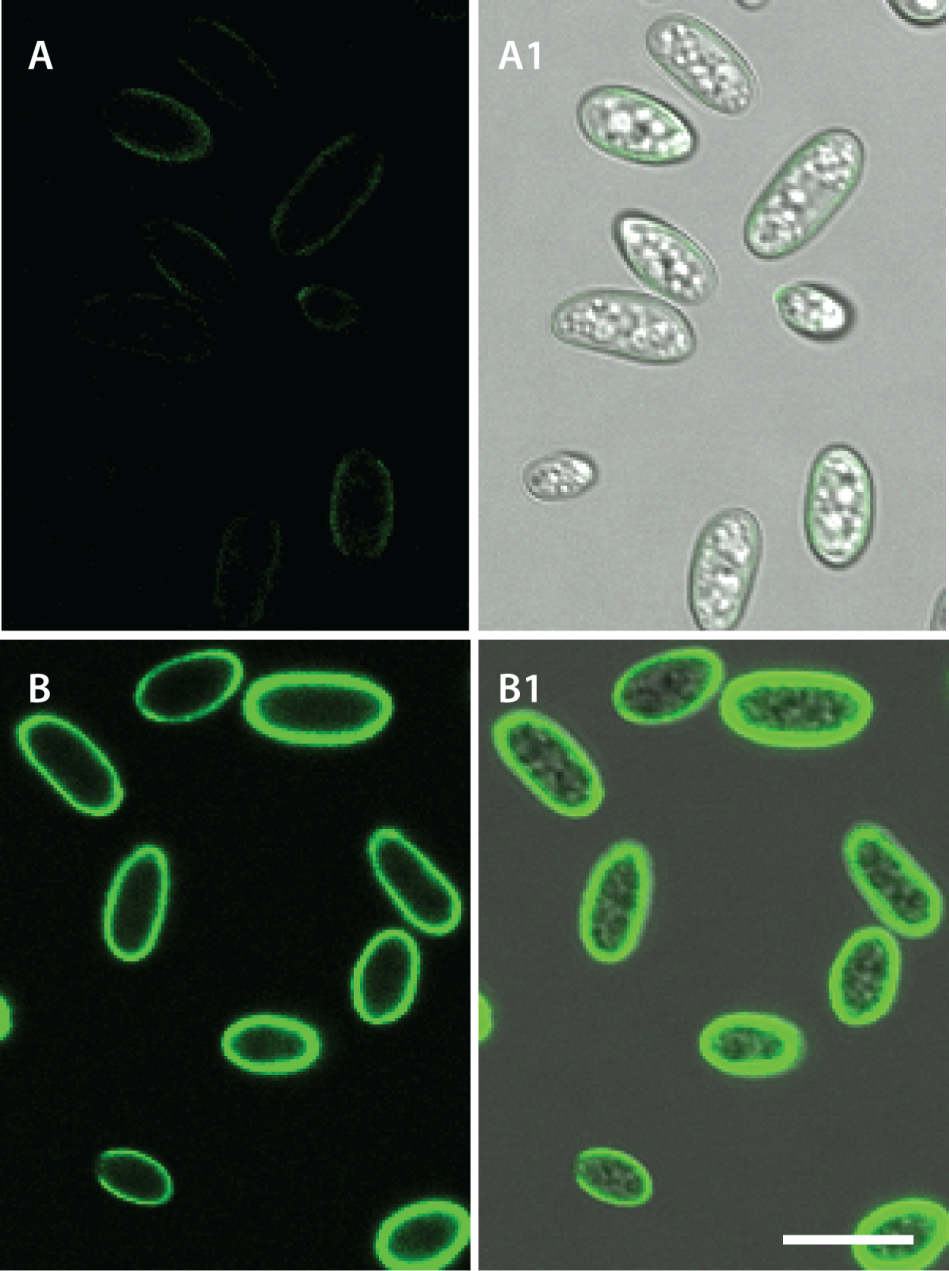
Cell wall specific staining of *F*. *oxysporum* microconidia, germlings and CAT fusion. (A, A1) Uniform staining of ungerminated, microconidial cell walls by Con A conjugated to Alexa fluor 488. (B, B1) Uniform staining of ungerminated, microconidia by WGA conjugated to Alexa fluor 488. A and B show fluorescence channels alone whilst A1 and B1 shows overlay images of the brightfield and fluorescence channels. Scale bar = 10 μm.

### Cytoplasmic connection and movement of organelles established by CATs

**Fig 14:**
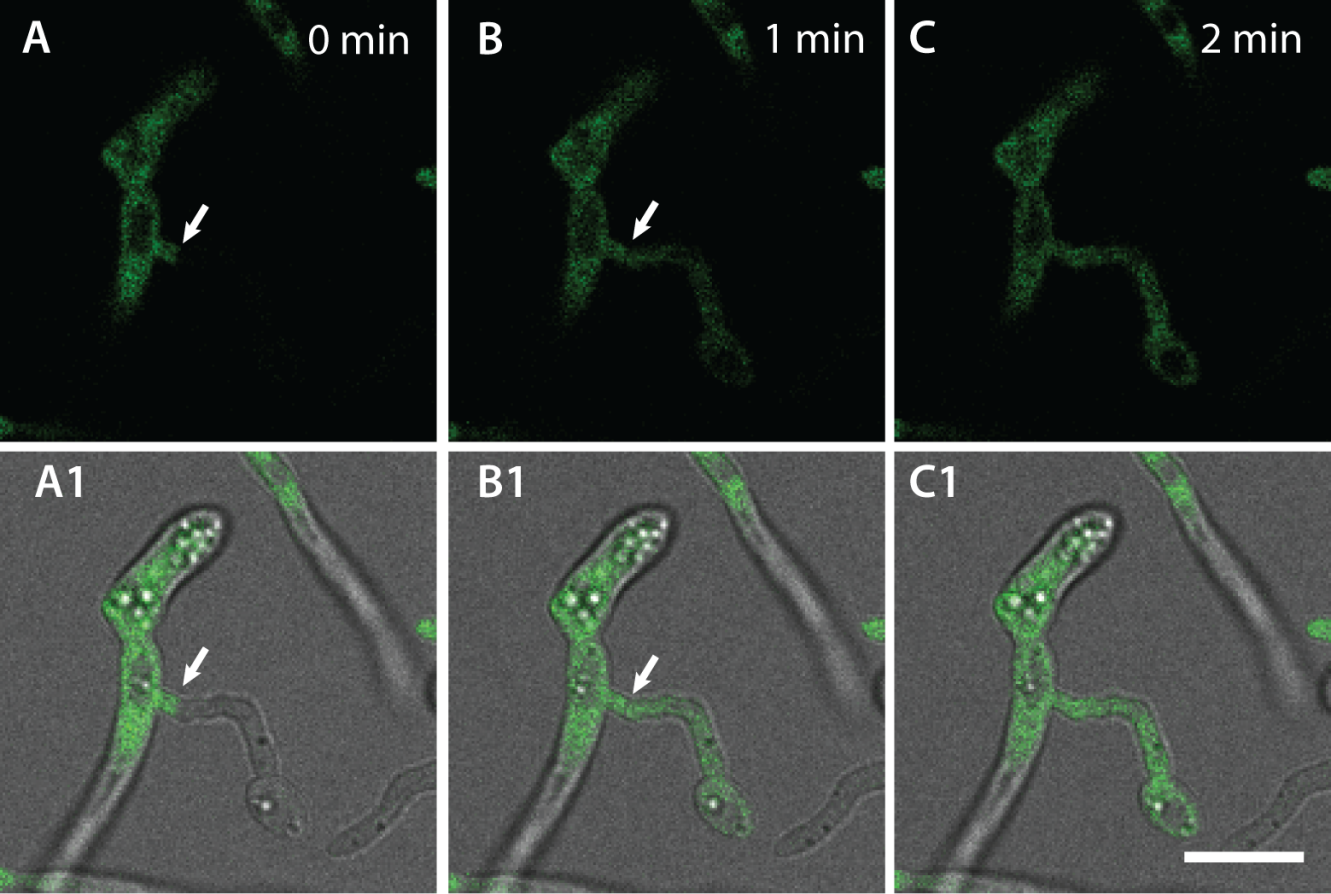
Cytoplasmic flow and mixing following CAT fusion. (A-C) Time course showing final stages of CAT fusion between a cytoplasmic GFP expressing strain (left) and its parental wild type strain (right). When the attached intervening cell walls of the two CATs became degraded, GFP moves from the left to the right hand germling indicating cytoplasmic mixing and continuity between the two germlings. A1, B1 and C1 shows overlay images of brightfield and fluorescence channels while A, B and C shows GFP channel alone. Arrows point to region of fusion. Scale bar = 10 μm.

**Fig 15:**
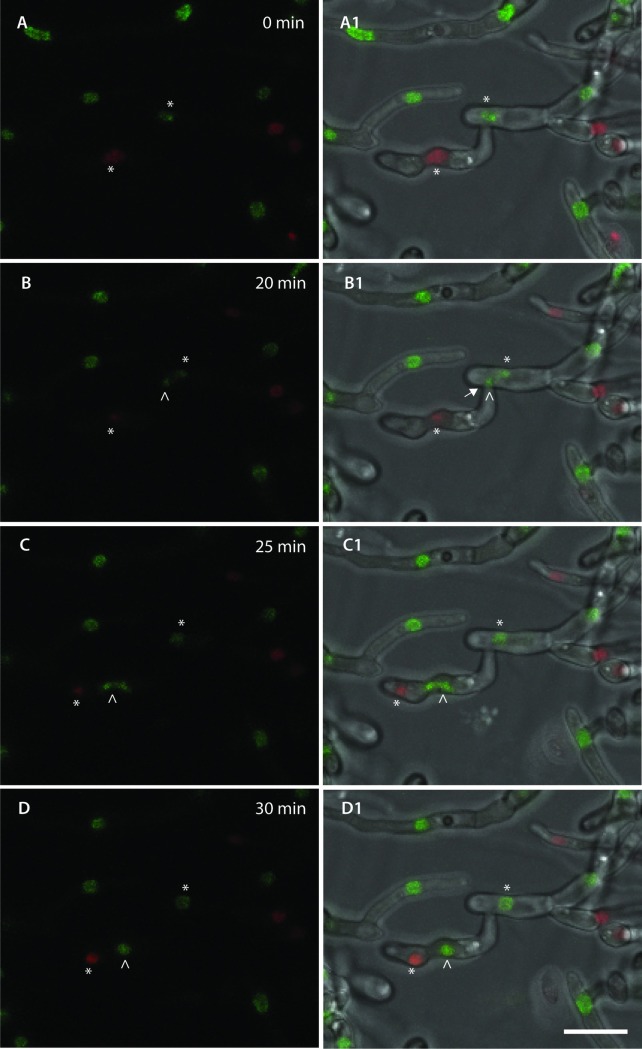
CAT fusion facilitates the movement of nuclei between fused germlings (see also Movie 4.5). (A-D) Time course of nuclear division and migration observed during CAT fusion between H1-GFP (green) and H1 mCherry (red) nuclear labelled strains imaged in an overlay of only the green and red fluorescence channels. (A1-D1) Corresponding images from the same time course imaged in an overlay of the two fluorescence channels and the brightfield channel. The point of cell wall fusion is indicated with an arrow in B1. Two nuclei, one in red and another in green are highlighted with asterisks. The green nucleus underwent a mitotic division at the 20-min time point (daughter nuclei highlighted with an empty arrowhead). At 25 min, one of the green daughter nuclei migrated through the site of CAT fusion (arrow in B1) to the cell compartment containing a red nucleus (C and C1). At 30 min, the green nucleus regained its spherical form and shared the same cell compartment as the red nucleus (D and D1). Scale bar = 10 μm.

**Fig 16:**
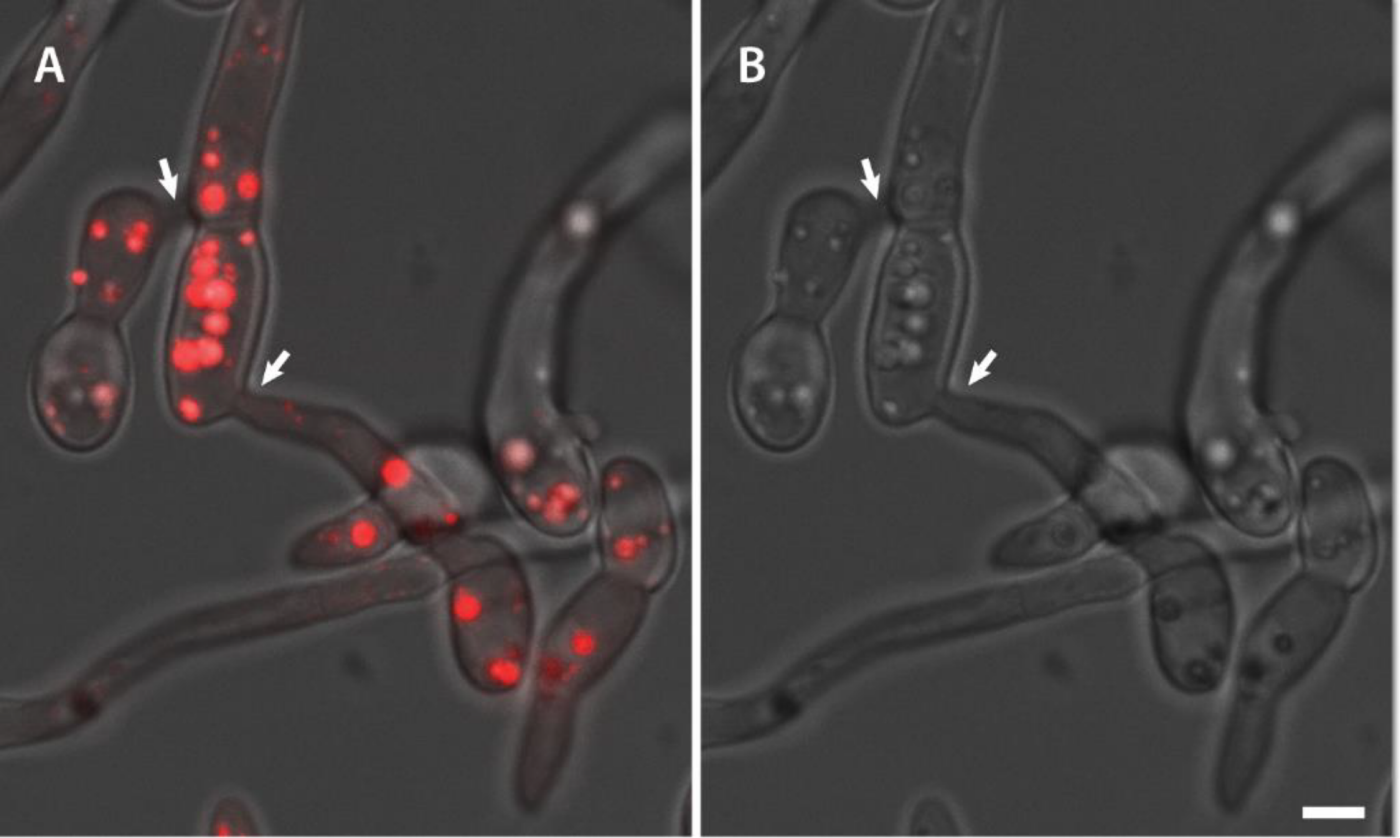
The refractile organelles within microconidia and germlings are lipid droplets. (A) The lipid-specific dye Nile red stains these organelles as shown in an overlay image of the fluorescence and brightfield channels. Two CAT fusions (arrows) can be seen between three germlings in the image. (B) Refractile lipid droplets visualised in the brightfield channel visualised in (A). Scale bar = 10 μm.

**Fig 17:**
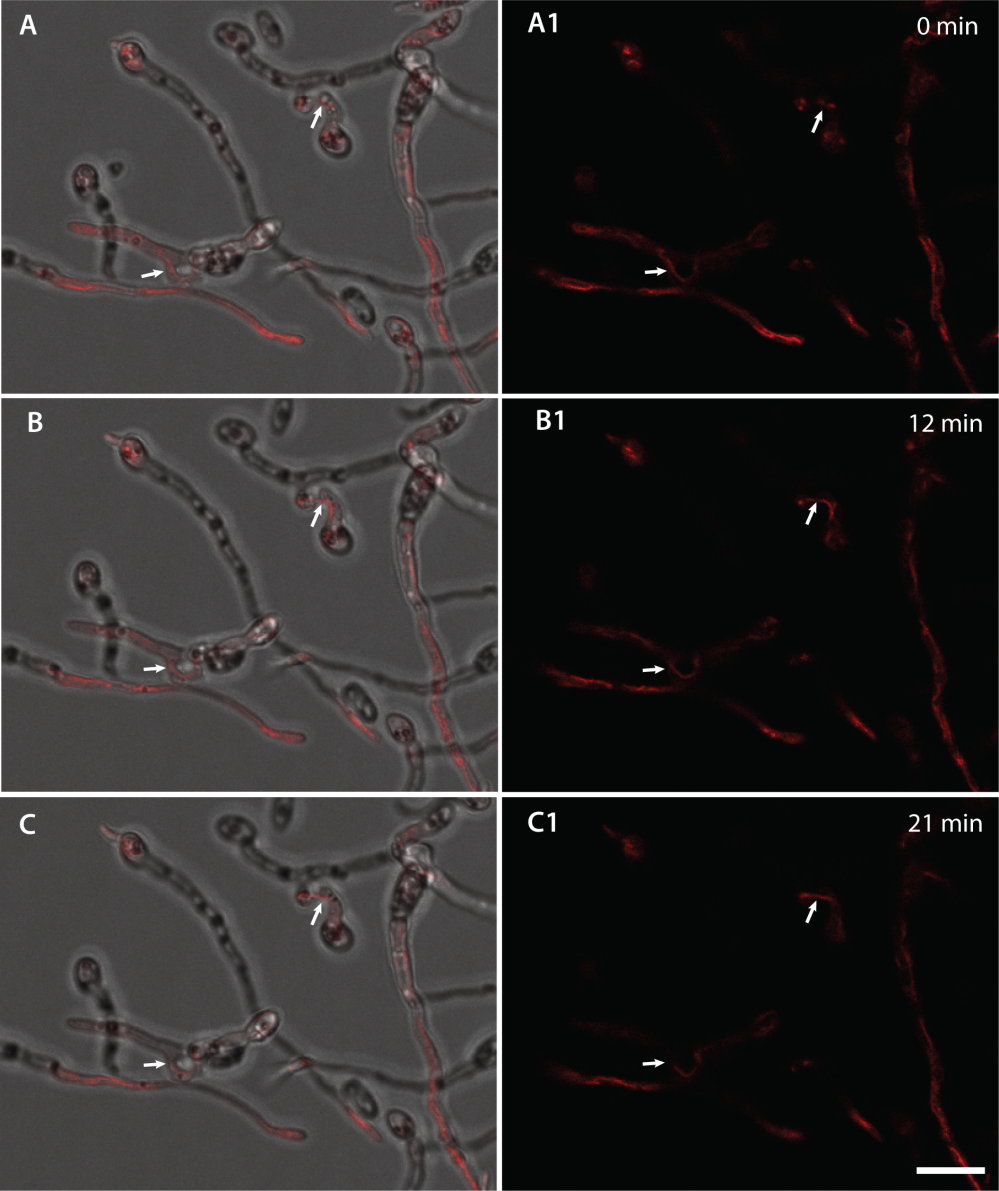
CAT fusion facilitates the movement of mitochondria between fused germlings. (A-C). Time course showing the movement of mitochondria through sites of CAT fusion (indicated by arrows) between germlings. Mitochondria were stained with the mitotracker red stain. A-C are overlays of brightfield and red fluorescence channels whilst A1-C1 is the red fluorescence channel alone. Scale bar = 10 μm.

**Fig 18:**
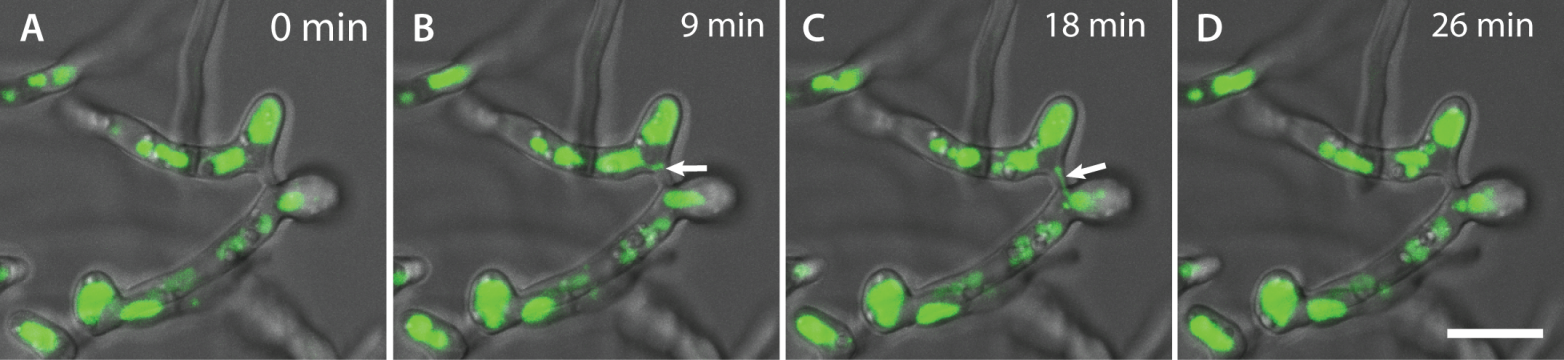
CAT fusion facilitates the movement of vacuoles between fused germlings. (A-D) Time course showing the movement of vacuoles between two germlings through CATs that have fused. At 9 min (B), a small vacuolar compartment (arrow) can be seen pinching off from a larger one, before it becomes more elongated as it passed through the fused CAT connection between the two germlings. At 18 min (C), the small vacuole can be seen moving through the fused CAT connection into the second hyphal compartment (C). The movement of the vacuole was completed at 26 min (D). The vacuoles were stained with the cDFFDA stain. Scale bar = 10 μm.

Live-cell imaging provided conclusive evidence of cytoplasmic connections being formed by CAT fusion (Fig 14). We observed the movement of nuclei (Fig 15; S10 Movie), refractile organelles (later shown to be lipid droplets, see Fig 16), mitochondria (Fig 17; S11 Movie) and vacuoles (Fig 18; S12 Movie) between the two fused germlings (Fig 7; S6 Movie). We analysed the cytoplasmic continuity of CAT fusions by mixing microconidia from a strain expressing cytoplasmic GFP with the unlabelled parental wild type strain from which it was derived. Live-cell imaging showed an immediate flow and mixing of cytoplasm between the germlings, once the intervening attached cell walls between fusing CAT had been degraded (Fig 14; S9 Movie).

Two strains expressing histone H1-GFP (green) or H1-mCherry (red), respectively were used to follow the movement of nuclei between and within fused germlings over a period of 30 min (Fig 15; S10 Movie). These observations demonstrated that nuclei moved through fused CATs, as previously described [19]. Mitotic division was commonly observed during this process (Fig 15; S10 Movie). In the example shown, the red nucleus was not degraded after migration of the green nucleus into the ‘host’ cell, as previously reported [19]. However, this may be due to the short time of the observation (only conducted over 30 min), since nuclear degradation was reported to take place several hours after the external nucleus has entered the host cell [19].

**Fig 19:**
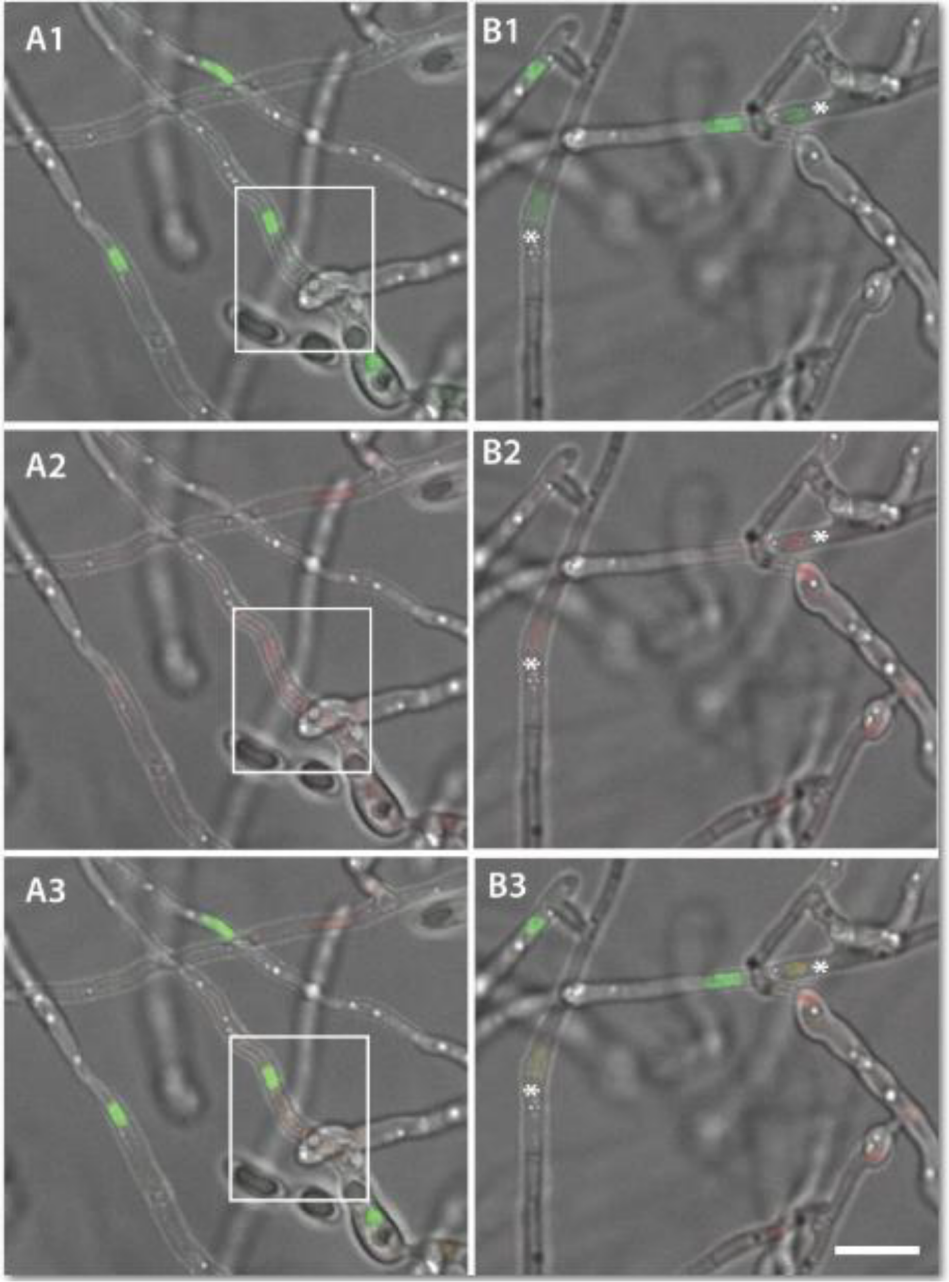
Time courses showing the fate of nuclei following CAT fusion. (A1-A3) One nucleus within the box is intact and green (A2) whilst another nucleus in the same hypha and within the box shows faint dispersed red fluorescence, indicative of degradation (A2). The overlay images show that there is no colocalization of the green and the faint red nucleus (A3). (B1-B3) Two nuclei (asterisk) containing both H1-GFP (B1) and H1-mCherry (B2) appear yellow in the overlay images (B3). A1-A3 and B1-B3 are overlays of the green fluorescence, red fluorescence and brightfield channels. H1-GFP and H1-mCherry strains were incubated for 72 h in 1% PDB supplemented with 25 mM NaNO_3_. Scale bar = 10 μm.

The strains expressing H1-GFP and H1-mCherry were imaged after co-incubation for 72 h. In these experiments, evidence of a red nucleus undergoing degradation whilst sharing the cell compartment with an intact, healthy looking green fluorescent nucleus was frequently observed following CAT fusion (Fig 19A1–19A3). This is consistent with the observations of Ruiz-Roldán *et al*. [19] although it was not possible to capture the process of nuclear migration from the ‘donor’ cell to a ‘host’ cell in a time course. We observed that cell fusions were more frequent after 72 h co-incubation. However, these were not CAT fusions between conidial germlings but between more mature hyphae and thus are referred to as vegetative hyphal fusions.

Another observation after incubating the red and green fluorescing strains for 72 h was the presence of red, green and yellow nuclei in some fields of view (Fig 19B1–19B3). However, because of the extensive hyphal growth that had occurred after 72 h, it was not possible to convincingly confirm that any of the cell compartments that contained a yellow nucleus were uninucleate, which would be indicative of a second nucleus having undergone degradation. In addition, no obvious increase in the volume of the yellow nuclei compared with the green and red nuclei was observed, which suggests that no fusion between green and red nuclei had occurred.

Mitotracker Red, a mitochondrial stain [32], was used for live-cell imaging of mitochondrial organization and dynamics during CAT fusion. Although difficult to observe, tubular mitochondria were found to move between germlings through fused CATs (Fig 17; S11 Movie).

cDFFDA, a vacuolar stain [32], was used for live-cell imaging of vacuolar organization and dynamics during CAT fusion. Round and elongated vacuoles of varying sizes and shapes were observed in germ tubes and CATs. The individual vacuoles and their morphologies were very dynamic when observed in time course movies. Small vacuoles pinching off from larger ones were observed to move through fused germlings (Fig 18; S12 Movie).

**Fig 20:**
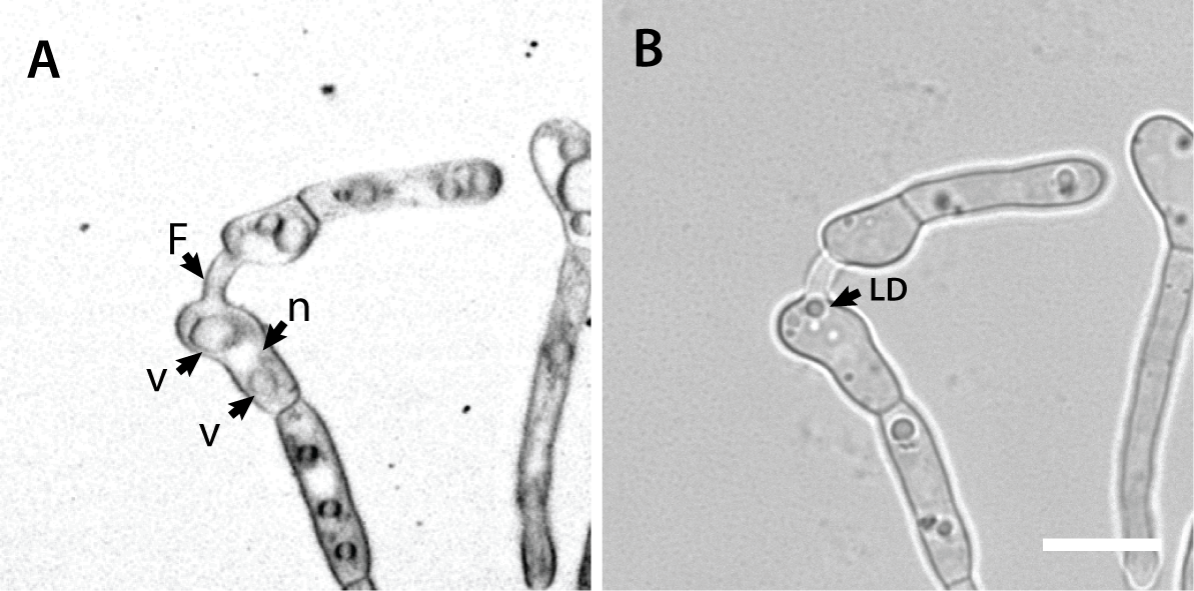
Lipid droplets are distinct from vacuoles and nuclei. (A) Negative image of the fluorescence image of two germlings that have undergone CAT fusion with each other (labelled as F), after staining with the membrane selective fluorescent dye FM4-64. The membranes of vacuoles (v) and nucleus (n) have been labelled. (B) Brightfield image of the same germlings shown in A, in which one of the refractile lipid droplets is highlighted (labelled as LD), which was unstained in the fluorescence image shown in (A). More such Refractile lipid droplets can also be seen in other cell compartments. Scale bar = 10 μm.

Previously unidentified, spherical refractile organelles of varying size (< 2.4 μm in width) were consistently observed in microconidia, germ tubes and CATs. Microconidia and germlings were commonly filled with these organelles, which were easily visualised by differential interference contrast or brightfield microscopy (e.g. S2, S5 and S6 Movies). The mitochondrial dye mitotracker red, the vacuolar dye cDFFDA or the membrane selective dye FM4-64 (Fig 20A) all failed to stain these organelles. However, Nile red, a neutral lipid selective dye [33], stained the round organelles strongly, indicating that they are lipid droplets. These droplets also moved between fused germlings through CATs (Fig 16; S1 Movie). However, the growing tips of germ tubes and CATs were devoid of these organelles as evident from live-cell imaging (S6 Movie).

## Discussion

An optimal set of conditions was determined for promoting *in vitro* CAT fusion of *F*. *oxysporum f*. *sp*. *lycopersici* strain Fol4287, whose genome has been sequenced. From these studies, the following standard growth medium and culture conditions were selected for routine quantitative analyses and live-cell imaging of spore germination and CAT fusion in 8-well slide culture chambers: unbuffered 1% PDB supplemented with 25 mM NaNO_3_, inoculated with 1 x 10^6^ spores/ml and incubated at 25°C for 12 h in continuous light. Some important observations were also made relating to spore and germling adhesion, the dependence of CAT fusion on cell adhesion to the substratum, the time course of spore germination and CAT fusion, and the influence of pH on spore germination and CAT fusion. Observations made by a detailed cytological analysis of the process of CAT fusion by live-cell imaging identified different types of CAT fusion, different stages of CAT fusion, differences in cell wall composition, differences between the widths of CATs and GTs, and the movement of organelles including nuclei, mitochondria, vacuoles and lipid droplets through fused CATs.

### Conidial and germling adhesion

Microconidia and germlings of *F*. *oxysporum* failed to adhere to the borosilicate glass substratum of the slide culture chamber when incubated in 1% PDB alone, whereas addition of NaNO_3_, CaCl_2_, NaCl, MgCl_2_, KCl, or glutamic acid triggered germling adhesion. Non-adhered microconidia or germlings exhibited Brownian movement in liquid media and thus were readily distinguished from adhered propagules that were immobilised on the substratum surface, as previously reported for conidia of *Phyllosticta ampelicida* [34]. After 6 h, only a minority of ungerminated conidia had adhered, but between 6 and 10.5 h an increasing number adhered, germinated to form single germ tubes and finally underwent CAT fusion. These results suggest that the surface properties of microconidia and germlings at 7.5 h are different from freshly harvested microconidia because the latter did not adhere to the glass. The time related to acquiring adhesive surface properties may play an important role in the achievement of developmental competence. However, how NaNO_3_ and the other chemicals induce cell adhesion is unclear. In the case of NaNO_3_, adhesion is unlikely to result from a nutritional role because it occurs very rapidly. We speculate that NaNO_3_ and the other chemicals play a critical role in influencing the charge properties of the spore and hyphal surfaces, causing them to become instantly adhesive to the glass substratum. Production of an extracellular matrix outside the fungal cell wall was found to be important for adhesion in different plant pathogenic fungi (e.g. [35, 36, 37]). In the presence of PDB, macroconidia of *F*. *solani* produce mucilage at the tips (so-called ‘spore tip mucilage’ or STM), allowing them to adhere to a polystyrene surface. STM was found to contain glycoproteins and α-mannans/α-glucans, and accordingly adhesion was blocked by adding the α-mannan/α-glucan binding lectin, concanavalin A (ConA) [35]. Although STM was not detected in microconidia upon staining with fluorescently labelled ConA [35], here we observed α-mannans on the surfaces of both microconidia and germ tubes (Fig 10). Similar results were also reported by Schoffelmeer *et al*. [30]. The outer layer of fungal cell walls is commonly rich in α-mannans, which are components of glycoproteins with a role in fungal cell adhesion [38, 39, 40]. However, it is unlikely that α-mannans play a role in the adhesion of *F*. *oxysporum* germlings because addition of Con A did not inhibit their adhesion (data not shown).

Hydrophobic interactions between the cell and the substratum have been shown to play a role in fungal cell adhesion. Notably, hydrophobins present on the surface of the cell wall, such as MPG1 and SC3, contribute to cell adhesion to hydrophobic surfaces [41, 42]. However, currently there is no evidence for a role of hydrophobins in cell adhesion of *Fusarium spp*. On the other hand, MAP kinase signalling may play a role in cell adhesion in *F*. *oxysporum* because a mutant defective in the MAP kinase 1 gene *fmk1* exhibited impaired microconidial germling attachment to tomato root surfaces [29].

### External conditions impact on germ tube formation and CAT fusion

An appropriate combination of external conditions is important for the successful colonisation of a host by a fungal pathogen. Spore germination *in vitro* is influenced by a variety of environmental factors, including the presence of other spores. External conditions also influence CAT fusion, and the optimal external conditions for CAT fusion may not be optimal for germ tube formation. For example, CAT fusion was inhibited in 100% PDB in *N*. *crassa* and evidence was obtained that tryptophan played a role in this inhibition [43]. Here we tested a range of external factors in order to optimise the culture conditions for quantitative analyses and live-cell imaging of *in vitro* spore germination and CAT fusion in *F*. *oxysporum*. Although microconidia germinated well in either 100% PDB or minimal medium, CAT fusion was absent. In previous studies, *F*. *oxysporum* was reported to undergo germling fusion in diluted (2%) PDB supplemented with 20 mM glutamic acid and 0.8% agarose [18]. We thus used diluted PDB (1%) supplemented with a range of supplements, including glutamic acid, resulting in reproducible CAT fusion.

Of particular interest was the question whether germling adhesion to the substratum is a pre-requisite for CAT fusion. Although fusion on a glass surface was only observed when PDB was supplemented with NaNO_3_, CaCl_2_, NaCl, MgCl_2_, KCl or glutamic acid, all of which also induced adhesion, no fusion was observed with NH_4_NO_3_ or (NH_4_)_2_SO_4,_ even though these supplements also induced adhesion. A likely reason for this is that the media containing either of these ammonium salts were highly acidic (initial pH ~ 4.0), a condition which is not conducive for CAT fusion. Previous studies in other fungal species, including *N*. *crassa*, have not provided evidence for cell adhesion as a requirement for CAT fusion.

The influence of temperature on microconidial germination and CAT fusion was also assessed. Interestingly, germ tube formation was significantly inhibited at high temperature (35°C) whilst CAT fusion occurred at a similar level at all three temperatures tested. Different optimum temperatures for different physiological or developmental processes have been previously reported for *Fusarium spp*. For example, Gupta *et al*. [44] found in *F*. *oxysporum* f. sp. *pisi* and *F*. *solani*, that while 28°C was optimal for colony growth, maximum sporulation occurred at 34°C. High temperature of 35°C was found to inhibit growth of *F*. *graminearum* and *F*. *culmorum* on different media [45, 46]. Landa *et al*. [47] reported that *Fusarium* wilt of chickpea was most severe at 25°C compared to 20°C and 30°C.

Self-inhibition of spore germination has been described in a number of fungal species, and a range of different germination inhibitors have been identified [48, 49]. In *F*. *oxysporum*, autoinhibition at high spore densities was previously reported, and the self-inhibitor was identified as nonanoic acid [50, 51]. Here we found that a concentration of 5 x 10^6^ spores per ml had an inhibitory effect on germination and CAT fusion, while 10^6^ microconidia per ml was optimal for CAT fusion in *F*. *oxysporum*. This is comparable with the macroconidial density reported to be optimal for CAT fusion in *N*. *crassa* [25]. In the latter study, evidence was presented that CAT formation may exhibit a form of quorum-sensing behaviour (i.e. involving a mechanism in which cells monitor their population density by releasing signaling molecules into the environment). The CAT inducer in *N*. *crassa* has not yet been identified (1). Here we speculate that the reduced cell fusion observed at low concentrations of microconidia (1 x 10^5^ spores/ml) is likely to be due to the increased spatial distance between the spores/germlings producing CATs. Analysis of 15 different time-course experiments of CAT induction, homing and fusion at 10^6^ microconidia per ml suggested that the maximum distance between germlings undergoing positive CAT tropisms towards was around 8 μm. In *N*. *crassa*, the maximum distance allowing CAT homing to be initiated was 15 μm [52].

Our data suggest that microconidial germination in unbuffered medium occurs at similar rates with pH values between 4 and 7, while CAT fusion occurs at a similar level between pH 5 and 9. Importantly, both processes are inhibited at pH values of 4 or below. In buffered medium, germination occurred at a high level between pH 3.5 and 8.3, while CAT fusion was maximal at pH 5.5 or 6.3. Similarly, in *N*. *crassa*, CAT fusion was optimal in medium buffered at pH 5 or 6 [43]. External pH was previously shown to influence spore germination, hyphal growth and sporulation in *Fusarium spp*. but the optimum pH for these processes varies depending on the process and the species or species isolate analysed [44, 53, 54, 55]. For example, chlamydospore germination in *F*. *oxysporum* f. sp. *cubense* was highest at pH 8, which correlated with the soil pH at which infection resulting from chlamydospore germination was most severe [54]. In *F*. *oxysporum* f. sp. *pisi* and *F*. *solani*, maximum growth was reported at pH 5.5 while pH 6.5 was optimal for sporulation [44].

Here we examined different media for inducing CAT fusion in *F*. *oxysporum*, in addition to those used in previous studies [18, 20]. We conclude that CAT fusion can be induced by multiple specific nutrients or chemicals in the environment, not just by carbon and nitrogen limitation as reported previously [14, 20]. It is possible that multiple environmental stimuli target the same or shared signalling components to induce CAT fusion. Inhibition of CAT formation in NH_4_NO_3_ or (NH_4_)_2_SO_4_ containing media is suggestive of a role for nitrogen signalling in this process and may also indicate a role for pH signaling in CAT formation. In support of this idea we observed that CAT fusion was inhibited in *F*. *oxysporum* under highly acidic conditions. In fungi, a conserved regulatory pathway governs pH-dependent expression of secreted proteins and adaptation to alkaline stress, operating via the PacC/Rim101 transcription factor [56]. *F*. *oxysporum* has acid and alkaline expressed genes. The *F*. *oxysporum* PacC orthologue negatively and positively regulates specific genes under acidic and alkaline conditions, respectively [57, 58]. In *F*. *oxysporum*, PacC positively regulates the expression of ENA1, an orthologue of *S*.*cerevisiae* and *N*.*crassa* ENA1, a gene expressed during alkaline conditions and in the presence of high Na^+^ [58]. The role of PacC in pH-dependence of CAT fusion is currently unknown and needs further experimentation.

### CATs are structurally different from germ tubes

As a result of a detailed analysis of time-lapse images, the three basic stages of CAT fusion (i.e. CAT formation, homing and fusion) that were originally defined in *N*. *crassa* [26], were also identified in *F*. *oxysporum*. The duration of the complete process from incipient CATs to completed CAT fusion resulting in cytoplasmic continuity between the fused microconidial germlings varied between 41 and 168 min (n = 15). Although to our knowledge no rigorous quantification of the time period of CAT fusion has been performed so far in *N*. *crassa*, inspection of published images and movies of this process [1, 26] suggested that it usually occurs within 45 min. Interestingly, we noted that *F*. *oxysporum* displays a significant period between initiation of microconidial germination (after 1–2 h) and CAT fusion (after 8 h). This contrasts markedly with *N*. *crassa* in which macroconidial germination and CAT fusion are initiated at approximately the same time (after 3–4 h, [26]). Thus, microconidia may require ~ 8 h following inoculation to become developmentally competent to form CATs.

In *N*. *crassa*, CATs can be distinguished from germ tubes by exhibiting positive tropism towards each other [26]. As mentioned above, the maximum initial distance between two CATs of *F*. *oxysporum* growing chemotropically towards each other was ~ 8 μm, compared to ~ 15 μm in *N*. *crassa* [52]. This presumably relates to the distance at which an appropriate concentration of the unidentified CAT inducer/chemoattractant can reach its ‘partner spore/germling’ to activate the yet unidentified mechanism which triggers communication via the CAT fusion self-signalling mechanism [52]. This parameter could be influenced by many factors, e.g. the amount of the CAT inducer/chemoattractant released from the CAT tip, the number of ligand receptors present at the receiver CAT tip, or the amount of ligand-degrading enzyme that might be secreted into the extracellular space in order to generate CAT inducer gradients between communicating cells.

We found that *F*. *oxysporum* CATs can usually be distinguished morphologically from GTs by being thinner on average (average CAT width = 1.73 ± 0.52 μm; average GT width = 2.45 ± 0.45 μm). In *N*. *crassa* CATs were also reported to be thinner than GT, although their average width varies between wild type strains [26]. *Fusarium oxysporum* CATs emerged either directly from microconidia or from a GT, either from the tip or as a subapical branch, as reported in *N*. *crassa* [26]. Different types of observed CAT fusion include tip-to-tip fusion of two CATs formed from growing GTs or from conidia, tip-to-side fusion of a CAT formed from a GT to a conidium, and side-to-side fusion of CATs from adjacent GTs. In some cases, CATs were not visible between fusing conidia/GTs that were immediately adjacent to each other. Similar types of CAT fusion were previously reported in *N*. *crassa* [26].

A newly discovered difference between CATs and GTs in *F*. *oxysporum* is the distinct cell wall surface composition, as demonstrated using fluorescently labelled lectins, which are selective for different cell wall surface polysaccharides. Our results demonstrate that the CAT surface is significantly enriched in α-mannans and α-glucans compared to that of GTs or microconidia. The polysaccharides may either be part of the cell surface itself, or of an extracellular matrix layer that is secreted at the surface of CATs. It is possible that the α-mannans and/or α-glucans play a role in cell adhesion. A second difference was that, in contrast to GTs and microconidia, CATs lacked surface-exposed chitin. Although, CAT cell walls still contained chitin as revealed by CFW staining, this polymer was distinctly absent from the CAT surface. Interestingly, small subapically concentrated patches of chitin on the surfaces of GT cell walls were observed. Schoffelmeer *et al*. [30] obtained similar results when staining microconidia and germ tubes of *F*. *oxysporum* with fluorescently labelled Con A, WGA and CFW, although they did not analyse the cell wall composition of CATs. Finally, an enrichment of chitin was also observed at the base microconidiation of sites along the GTs.

Spore germination is accompanied by the synthesis of new wall material concomitant with the emergence of a GT [59]. A major difference in cell surface composition was observed in relation to chitin, because microconidia stained more intensely with fluorescently labelled WGA than GTs, and they lacked the spot-like staining observed on GTs. Multiple patches of concentrated chitin have been observed in ungerminated macroconidia *F*. *graminearum* using fluorescently labelled WGA [60].

### Organelles move between germlings via CAT fusions

Nuclei were previously shown to move between fused germlings of the plant pathogen *Colletotrichum lindemuthianum* [61]. By performing time-lapse live-cell imaging of labelled organelles, we observed the movement of nuclei, mitochondria, vacuoles and lipid droplets through the connections between germlings created by CAT fusion. While nuclear movement between fused germlings was described previously [18], the movement of mitochondria, vacuoles and lipid droplets has not been documented so far by live-cell imaging in *F*. *oxysporum*.

As previously described [18], we observed in liquid media conditions, events of nuclear degradation in the cell where a ‘donor’ nucleus had migrated following fusion, although it was not possible to image the entire process by time-lapse imaging. Such events of nuclear degradation have been proposed to favour horizontal chromosome transfer among incompatible strains, involving the incorporation of genetic elements from the degraded to the surviving nucleus [62]. However, compatible strains (both derived from Fol4287 expressing H1-GFP or H1-RFP) were used in our study. Vlaardingerbroek *et al*. [16] suggested that nuclear degradation may be involved in the selective loss of chromosomal regions. Examples of yellow nuclei were also observed in our study, although it was not possible to obtain unequivocal evidence that these nuclei were located in uninucleate cell compartments because of the extensive hyphal growth that had occurred after 72 h. It is thus possible that these yellow nuclei resulted from green H1-GFP and red H1-RFP expressing nuclei sharing the same cytoplasm and thus sharing their fluorescent fusion proteins. No morphological evidence was obtained for yellow nuclei being diploids, resulting from the fusion between a green and red nucleus, since no clear increase in the volume of yellow nuclei compared with the green and red nuclei was observed.

The observation of multiple fusion events and movement of cytoplasm and organelles between germlings via CAT fusion strongly suggests a role for CAT fusion in facilitating the re-distribution of cell contents within the mycelium, thereby achieving a state of a homogenous stable colony. This is in contrast to what has been reported by Shahi *et al*. [20] who rarely observed CAT fusion between multiple germlings of *F*. *oxysporum*. However, the media conditions and times of analysis differed between their study and ours. Increased mixing of nuclei could improve horizontal transfer of genetic material, which might be a strategy of selective advantage to adapt to stress conditions such as a nutrient limitation.

Considering the significance of *F*. *oxysporum* as a major plant and animal pathogen [5, 6, 63], it is important to understand the early developmental stages from microconidia, which can serve as inocula for infection [64, 65]. While much is known about the molecular and genetic mechanisms underpinning virulence [66], detailed studies linking these data to the morphology of conidial development by advanced microscopy are lacking. Our observations made by live-cell imaging have contributed to visualise the process of conidial germination and CAT fusion *in vitro*, revealing new insights into the concomitant morphological changes. Further studies are required to elucidate the intricate details and underlying genetic regulation of these morphogenetic events.

## Supporting information

S1 Movie Germination of microconidia of *F*. *oxysporum* in 1% PDB. Only germ tubes are formed and the germlings do not undergo adhesion to the underlying borosilicate glass substratum nor do they exhibit CAT fusion. The spores were incubated for 12 h at 25°C and a time course over 60 sec was generated with an image captured every sec. The frame rate of the movie is 7 frames/sec. Scale bar = 10 μm.
(MP4)

S2 Movie Germ tube formation, cell adhesion and CAT fusion in 1% PDB supplemented with 25 mM NaNO_3_. The spores exhibit Brownian movement in the liquid media prior to adhesion. Only a few ungerminated spores had adhered, whilst all the germlings had adhered at this time point. Germlings undergoing CAT fusion are highlighted in the white circles. The spores were incubated for ~ 6 h at 25°C. The time course was generated over 233 min with an image captured every 30 sec. The frame rate of the movie is 12 frames/sec. Scale bar = 10 μm.
(MP4)

S3 Movie CAT fusion is induced in 1% PDB supplemented with 25 mM NaNO_3_. The germlings had adhered to the borosilicate glass substrate prior to undergoing CAT fusion. The spores were incubated for 12 h at 25°C. The time course was generated over 60 sec with an image captured every sec. The frame rate of the movie is 7 frames/sec. Scale bar = 10 μm.
(MP4)

S4 Movie Microconidial germlings undergo strong adhesion to substrate prior to CAT fusion. Addition of water droplets from a 200 μl pipette during imaging failed to displace adhered microconidial germlings incubated in 1% PDB supplemented with 25 mM NaNO_3_ for 12 h at 25°C. The time course was generated over 60 sec with an image captured every sec. The frame rate of the movie is 12 frames/sec. Scale bar = 10 μm.
(MP4)

S5 Movie Addition of specific compounds induce adhesion of germlings in media. Adhesion of microconidial germlings in 1% PDB alone was immediately induced when drops of 50 μg/ml NaNO_3_ were added from a pipette at the 82 sec time point after incubation for 7.5 h at 25°C. The time course was generated with an image captured every 10 sec. The frame rate of the movie is 20 frames/sec. Scale bar = 10 μm.
(MP4)

S6 Movie Stages of CAT fusion in *F*. *oxysporum*. CAT induction was the first stage, seen as small projections arising from the tip of the upper germ tube and side of the tip of the lower germ tube (0 min). This was followed by CAT homing indicated by the two CATs growing towards each other (0 to 53 min), CAT fusion when the walls of the projections are attached (53 min) and then degraded to establish a cytoplasmic connection between the fused CATs (54.0–54.5 min). The movement of refractile lipid droplets through the fused CATs can also be seen. The spores and germlings were incubated at room temperature in 1% PDB supplemented with 25 mM NaNO_3._ The time course was generated with an image captured every 30 sec over 112 min. The frame rate of the movie is 7 frames/sec. Scale bar = 10 μm.
(MP4)

S7 Movie Germlings displaced during CAT fusion. Two germlings that were initially touching each other became displaced as one or both CATs grew between the cells resulting in the germlings being pushed apart. Although it was not possible to visualise, cytoplasmic continuity between the two germlings, this was probably achieved at some point during the germling displacement process. The spores were incubated at room temperature in 1% PDB supplemented with 25 mM NaNO_3._ The time course was generated by capturing an image every 30 sec over 170 min. The frame rate of the movie is 20 frames/sec. Scale bar = 10 μm.
(MP4)

S8 Movie CAT fusion shows selectively brighter staining with Con A. Time course showing the bright staining of sites of CAT fusion stained with Con A conjugated to Alexa fluor 488. The spores and germlings were incubated at room temperature in 1% PDB supplemented with 25 mM NaNO_3._ The time course was generated by capturing an image every 3 min over 1 h 53 min. The frame rate of the movie is 7 frames/sec. Scale bar = 10 μm.
(MP4)

S9 Movie CAT fusion facilitates cytoplasmic streaming between fused germlings. Time course showing final stages of CAT fusion between a cytoplasmic GFP expressing strain (left) and its parental wild type strain (right). When the attached intervening cell walls of the two CATs became degraded, GFP flowed from the left hand to the right hand germling indicating cytoplasmic mixing between the two germlings. Overlay of brightfield and fluorescence channels have been shown here. The spores/germlings were incubated at room temperature in 1% PDB supplemented with 25 mM NaNO_3._ The time course was generated from images captured every min over 2 min. The frame rate of the movie is 1 frame/sec.
(MP4)

S10 Movie CAT fusion facilitates the movement of nuclei. CAT fusion facilitates the movement of nuclei between fused germlings. Time course of nuclear division and migration observed during CAT fusion between H1-GFP (red) and H1 mCherry (red) nuclear labelled strains imaged in an overlay of the green, red and fluorescence channels. The green nucleus in the centre of field of view undergoes mitotic division at the 20 min time point. This is followed at 25 min by one of the green daughter nuclei from this division migrating through the site of CAT fusion to the cell compartment containing a red nucleus. At 30 min, the green daughter nucleus that had migrated regained its spherical form and shared the same cell compartment as the red nucleus. The spores/germlings were incubated at room temperature in 1% PDB supplemented with 25 mM NaNO_3._ The time course was generated by capturing an image every 5 min over 2 h and 15 min. The frame rate of the movie is 30 frames/sec.
(MP4)

S11 Movie CAT fusion facilitates the movement of mitochondria. Time course showing the movement of mitochondria through two sites of CAT fusion between germlings (see Fig 17). Mitochondria were stained with the mitotracker red stain. Overlay of the brightfield and fluorescence channels. The spores/germlings were incubated at room temperature in 1% PDB supplemented with 25 mM NaNO_3_ for 12 h. The time course was generated with an image captured every 3 min over 38 min. The frame rate of the movie is 7 frames/sec.
(MP4)

S12 Movie CAT fusion facilitates the movement of vacuoles. CAT fusion facilitates the movement of vacuoles between germlings that had undergone CAT fusion (see Fig 18). The vacuoles were stained with cDFFDA. The spores/germlings were incubated at room temperature in 1% PDB supplemented with 25 mM NaNO_3._ The time course was generated by capturing an image every 3 min over 1 h 12 min. The frame rate of the movie is 7 frames/sec.
(MP4)
